# Mycobiome of Cysts of the Soybean Cyst Nematode Under Long Term Crop Rotation

**DOI:** 10.3389/fmicb.2018.00386

**Published:** 2018-03-16

**Authors:** Weiming Hu, Noah Strom, Deepak Haarith, Senyu Chen, Kathryn E. Bushley

**Affiliations:** ^1^Department of Plant and Microbial Biology, University of Minnesota Twin Cities, Saint Paul, MN, United States; ^2^Department of Plant Pathology, University of Minnesota Twin Cities, Saint Paul, MN, United States; ^3^Southern Research and Outreach Center, University of Minnesota, Waseca, MN, United States

**Keywords:** soybean cyst nematode, *Heterodera glycines*, nematophagous fungi, arbuscular mycorrhizal fungi, Mortierellomycotina, crop rotation, next generation sequencing

## Abstract

The soybean cyst nematode (SCN), *Heterodera glycines* Ichinohe (Phylum Nematoda), is a major pathogen of soybean. It causes substantial yield losses worldwide and is difficult to control because the cyst protects the eggs which can remain viable for nearly a decade. Crop rotation with non-host crops and use of biocontrol organisms such as fungi and bacteria offer promising approaches, but remain hampered by lack of knowledge of the biology of nematode parasitic organisms. We used a high-throughput metabarcoding approach to characterize fungal communities associated with the SCN cyst, a microenvironment in soil that may harbor both nematode parasites and plant pathogens. SCN cysts were collected from a long-term crop rotation experiment in Southeastern Minnesota at three time points over two growing seasons to characterize diversity of fungi inhabiting cysts and to examine how crop rotation and seasonal variation affects fungal communities. A majority of fungi in cysts belonged to Ascomycota and Basidiomycota, but the presence of several early diverging fungal subphyla thought to be primarily plant and litter associated, including Mortierellomycotina and Glomeromycotina (e.g., arbuscular mycorrhizal fungi), suggests a possible role as nematode egg parasites. Species richness varied by both crop rotation and season and was higher in early years of crop rotation and in fall at the end of the growing season. Crop rotation and season also impacted fungal community composition and identified several classes of fungi, including Eurotiomycetes, Sordariomycetes, and Orbiliomycetes (e.g., nematode trapping fungi), with higher relative abundance in early soybean rotations. The relative abundance of several genera was correlated with increasing years of soybean. Fungal communities also varied by season and were most divergent at midseason. The percentage of OTUs assigned to Mortierellomycotina_cls_Incertae_sedis and Sordariomycetes increased at midseason, while Orbiliomycetes decreased at midseason, and Glomeromycetes increased in fall. Ecological guilds of fungi containing an animal-pathogen lifestyle, as well as potential egg-parasitic taxa previously isolated from parasitized SCN eggs, increased at midseason. The animal pathogen guilds included known (e.g., *Pochonia chlamydosporia*) and new candidate biocontrol organisms. This research advances knowledge of the ecology of nematophagous fungi in agroecosystems and their use as biocontrol agents of the SCN.

## Introduction

The soybean cyst nematode (SCN), *Heterodera glycines* Ichinohe (Phylum Nematoda), is one of the most devastating pathogens of the important agricultural crop soybean [*Glycine max* (L.) Merr] worldwide (Wrather et al., 2003). A report in 2005 declared more than 1 billion US$ in soybean yield loss were caused by SCN in 28 soybean producing states across the United States (Wrather and Koenning, 2006). Planting resistant cultivars is a common strategy to manage the SCN (Niblack and Chen, 2004). However, because of limited sources of resistance, the SCN has already begun to overcome resistance in most cultivars (Niblack et al., 2008). Crop rotation with non-hosts is considered to be one of the most effective management strategies (Niblack and Chen, 2004). Among the non-host rotation crops, corn (*Zea mays* L.) is the most common one. In 2016, corn and soybean dominated nearly two thirds of the farmland operated in midwestern US states like Minnesota (USDA, 2014). Previous studies have shown that annual rotation increases the yield of both corn and soybean as compared to monoculture, and suggested that the increase in soybean yield may be partially attributed to the decline of SCN populations during the corn rotation (Chen et al., 2001b; Grabau and Chen, 2016a).

**Figure 1:**
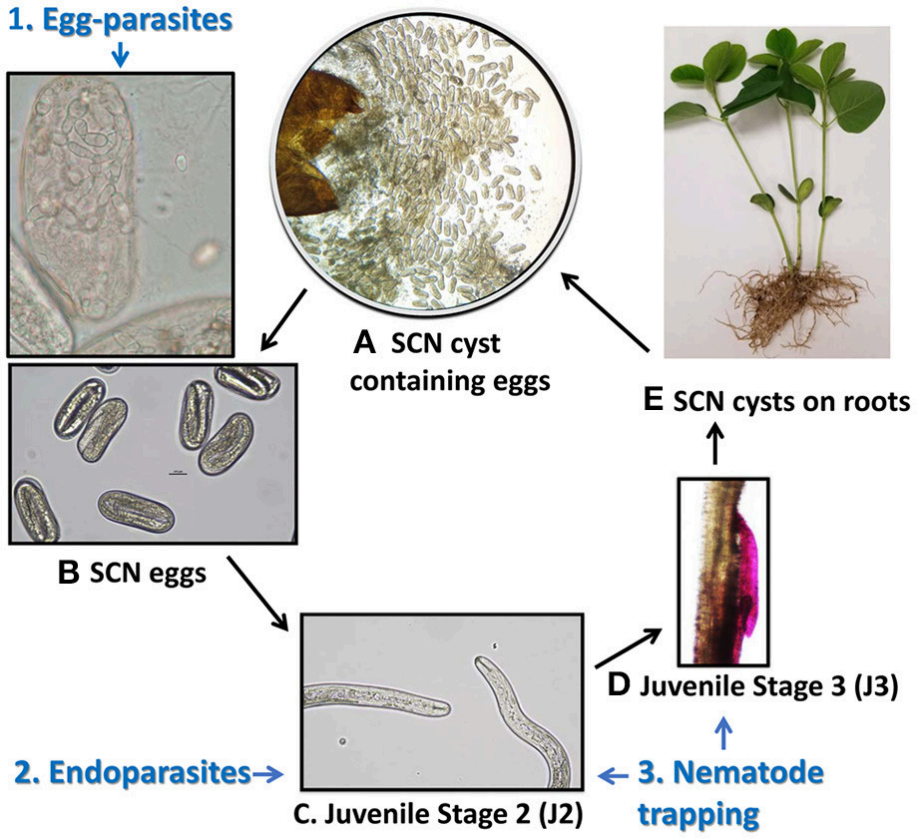
Life cycle of the SCN and life stage targeted by nematophagous fungi. **(A)** A cyst broken open to reveal hundreds of eggs inside, **(B)** Eggs each containing a juvenile nematode larvae, **(C)** Hatched juvenile stage 2 (J2) nematode larvae move through soil toward roots, **(D)** A juvenile stage 3 (J3) nematode larvae penetrating soybean root and forming a feeding site, and **(E)** fertilized females develop into cysts on soybean roots and may fall off into surrounding soil when mature. Different guilds of nematophagous fungi target distinct stages of the SCN life cycles. (1) egg-parasites infect eggs within cysts, shown here are hyphae invading an egg and sporulating, (2) endoparasites infect the mobile J2 in soil, and (3) nematode trapping fungi form elaborate hyphal structures to trap nematodes in soil or in rhizosphere surrounding the root.

However, a challenge in managing SCN is that it is nearly impossible to eradicate from soil once it becomes established in a field. The life cycle of SCN, which includes four developmental juvenile stages (J1-J4) and the adult male or female stage, is about 30 days under optimal conditions (Koenning, 2004; Figure 1). The second-stage juvenile (J2) nematodes infect soybean roots, forming feeding structures known as syncytia, to complete their life cycle. The SCN cyst forms from the melanized dead body of the fertilized female and can contain hundreds (ranging from ~100 to 600) of eggs. In a single growing season, the SCN can complete up to five life cycles. The cyst provides a protective environment for eggs, which will hatch under conducive conditions, but can also remain dormant and viable for nearly a decade when conditions do not favor hatching (Koenning, 2004; Chen, 2011). Thus, once present in soil, SCN eggs within cysts can remain viable far beyond the standard crop rotation schedules. The SCN cysts could also serve as a reservoir for various plant-pathogenic fungi, including important soybean pathogens such as *Fusarium virguliforme*, the causal organism of soybean sudden death syndrome (SDS) (McLean and Lawrence, 1995).

There is limited research on microbial communities in soybean crop rotation systems. Previous studies have shown that crop rotation impacts the structure of the total microbial community in bulk soil based on phospholipid fatty acid analysis (Vargas Gil et al., 2011). Differences in fungal community composition in bulk soil under soybean monoculture were also detected using denaturing gradient gel electrophoresis (DGGE), but this method was not sufficient to distinguish differences in diversity and structure of the microbial community (Li et al., 2010). Metabarcoding approaches using high-throughput sequencing offer the opportunity to more thoroughly characterize the impact of crop-rotation on microbial communities. One such study in wheat-soybean crop rotations showed that the bacterial community was impacted by different crop sequences (Yin et al., 2010). Some studies have suggested that crop rotation increases the diversity of the microbial communities compared to monoculture (Yao et al., 2006), while another showed that crop rotation reduced the abundance of potential plant pathogens, and increased plant growth promoting microbes (Zhou et al., 2017).

Microbial communities associated with the SCN, including fungi and bacteria inhabiting the microenvironment of the SCN cysts, are even less well-characterized. A study on bacterial communities found that the length of time of soybean monoculture led to distinct taxonomic differences between communities found inside SCN cysts from short term (<8 years) versus long term (>8 years) soybean monoculture (Zhu et al., 2013). Fungi found associated with the SCN include not only saprotrophs but also nematode pathogenic (nematophagous) fungi that offer potential for biocontrol of the SCN. Nematophagous fungi can be classified into three major groups based on the mechanism they use to kill nematodes during different stages of the SCN life cycle (Liu et al., 2009): (1) Nematode-trapping fungi that use specialized structures (e.g., sticky hyphae or knobs, hyphal nets, or constricting rings) to trap mobile juvenile stage 2 nematodes (J2) (Swe et al., 2011), (2) Endoparasites that are generally obligate parasites whose spores penetrate and consume J2 (Liu and Chen, 2000), and (3) Egg-parasitic fungi that parasitize eggs and sometimes mature females (Chen et al., 1996; Liu et al., 2009) (Figure 1). An early culture-based study on fungi suggested that parasitism of SCN by the J2-endoparasites was higher in soybean plots than corn plots in a soybean-corn rotation (Chen and Liu, 2007) and more recent research on SCN suppressive soil has found that potential SCN biological control egg-parasitic fungi such as *Pochonia chlamydosporia* (Syn. *Metacordyceps chlamydosporia*), *Exophiala* spp., and *Clonostachys rosea*, were more abundant in cysts from long-term monoculture plots (Hu et al., 2017). These egg-parasites offer particularly attractive candidates for biocontrol as they have potential to reduce inoculum of this pathogen in soil.

Although numerous studies have documented the effects of crop rotation on microbial communities in various microenvironments within agroecosystems including rhizoplane (Alvey et al., 2003), rhizosphere (Smalla et al., 2001), and bulk soil (Yin et al., 2010), the effects of crop rotation on fungal communities associated within SCN cysts under long-term crop rotation remains unknown. With the emergence of high-throughput sequencing technologies, there is growing opportunity to explore the microbial communities inhabiting the microenvironment of the SCN cyst in order to better understand the effects of crop rotation on both populations of the SCN and their associated fungal communities. Here, we used a high throughput (a.k.a. environmental metabarcoding) approach to characterize fungal communities in SCN cysts collected from a long-term rotation field site that has been in soybean-corn rotation for over 30 years and in which previous research shows that the SCN was the most important nematode pathogen affecting soybean yield (Grabau and Chen, 2016a). As some nematode parasitic fungi may follow a density-dependent population dynamic in which numbers of nematode parasites increases with increasing abundance of their nematode hosts (Jaffee, 1993), we also examined relationships between crop rotations, SCN egg densities, and fungal communities. We hypothesized that crop-rotation would lead to overall higher diversity of fungal communities than monoculture and that SCN fungal parasites would be positively correlated with both increasing years of soybean monoculture and SCN egg density. Thus, the objectives of this research were to characterize the key taxonomic and functional groups of fungi associated with SCN cysts and to document how these fungal communities within cysts vary by crop rotation, season, and SCN egg density in soil in order to advance the use of egg-parasitic fungi for biological control of the SCN.

## Materials and methods

### Field experimental design and management

The study was conducted at the Southern Research and Outreach Center in Waseca, MN, (44°04′N, 93°33′W) on a Nicollet clay loam (fine-loamy, mixed, mesic Aquic Hapludoll). At this field site, plots of various corn–soybean crop sequence treatments (annual rotation, 5 year rotation, and continuous monoculture) have been maintained continuously since 1982. The detailed experimental design of this field can be found in a previous report (Grabau and Chen, 2016b).

This study focuses on the fungal communities in the cysts of SCN during three seasons over 2 years in 2015 and 2016. The three seasons were spring (within 1 week before planting, May), midseason (47–64 days after planting, July), and fall (harvest season, late September or early October). The 10 crop sequences were as follows: (i) following 5 years of corn, treatments of 1, 2, 3, 4, and 5 years of soybean monoculture (S1, S2, S3, S4, S5), (ii) following 5 years of soybean, treatments of 1 and 2 years of corn monoculture (C1, C2), (iii) annual soybean-corn rotation with both cultivars (Ca, Sa), and (iv) soybean monoculture since 1982 (Ss). BT corn (DKC50-82 RIB) was used for all corn crop, and SCN-susceptible soybean (Pioneer P91Y90) was used for all soybean plots (Table 1). The crop sequence treatment was a complete block design with four replicates per crop sequence treatment.

**Table 1:** Soybean (S) and corn (C) cropping sequence treatments^†^ in Waseca, MN.

**Treatments**	**Crop sequence by year**	
	**2015**	**2016**
1	S2	S3
2	S3	S4
3	S4	S5
4	S5	C1
5	C1	C2
6	C2	C3
7	C3	C4
8	C4	C5
9	C5	S1
10	S1	S2
11	Ca	Sa
12	Sa	Ca
13	Ss	Ss
14	Sr	Sr
15	Cc	Cc
16	Cr	Cr

† *S1 through S5 are first- to fifth-year soybean after 5 year of corn; C1 through C5 are first- to fifth-year corn after 5 year of soybean; Sa and Ca are soybean-corn annual rotation with soybean and corn in the present year, respectively; Ss and Sr are continuous soybean with soybean cyst nematode (SCN)-susceptible and resistant cultivars since 2010, respectively. Cc and Cr are continuous corn with BT corn and non-BT corn (DeKalb DKC46-18), respectively. All soybean, except Sr, were susceptible to SCN. Treatments S1-S5, Sa, Ss, Ca, C1-2, and Cc were analyzed in this study*.

Plots were managed with conventional tillage using a field chisel to plow each fall and each spring before planting. Crops were fertilized to minimize the impact of soil nutrients on crop yield in this experiment. Nitrogen was applied at 224.4 kg/ha in the corn plots. All crops were Roundup-resistant, and glyphosate (Roundup) herbicide was used for pre-emergence and post-emergence weed control. Endigo Insecticide (for aphids), containing the insecticides thiamethoxam and lambda-cyhalothrinm, was sprayed at 245 g/ha at midseason.

### Soil sampling for egg population estimation

Soil samples were taken from the two center rows of each six-row plot (7.62 m long, 3.81 m wide); 20 soil cores were taken at a depth of 20 cm within 4 cm of plant growth. The soil cores were homogenized by passing through a sieve with 4 mm apertures. A subsample of 100 cm^3^ soil was taken from the soil samples and the cysts from the soil were extracted with a modified sucrose suspension and centrifuge method (Jenkins, 1964). The eggs were released from the cysts using a mechanical device (Faghihi and Ferris, 2000). The eggs were separated from the debris by centrifuging in 38% (w/v) sucrose solution, and collected from the top of the sucrose suspension in 25 ml water. The eggs were determined by counting a subsample of the egg suspension under an inverted microscope.

### Cysts sampling and collection

Soil samples were collected from the 2nd and 4th rows of each six-row plot. Cysts were extracted from the soil with a modified hand-decanting method (Chen et al., 2001c). About 4 kg of soil was soaked for 1 h in a bucket and stirred with an electric drill stirrer to break up soil aggregates. The soil suspension was then suspended with a strong jet of water, allowed to settle for 3–5 s to allow large soil particles and debris to settle, and then the upper layer of water containing cysts was poured through an 850-μm-aperture sieve nested on top of a 250-μm-aperture sieve. This procedure was repeated at least three times for each soil sample to each bucket of soil to collect the cysts in the soil sample. Cysts with debris and soil particles on the 250-μm-aperture sieve were collected, and the cysts were separated from the debris and soil particles with a sucrose flotation and centrifugation method (Jenkins, 1964) in 63% (w/v) sucrose solution. A subsample of 50 intact fully mature brown cysts were hand-picked randomly with forceps under an inverted microscope and treated with 0.5% NaOCl for 3 min to surface sterilize the cysts, then rinsed with sterile deionized water three times, and stored at −80°C until DNA extraction. To determine degree of parasitism, another subsample of 50 intact cysts were picked, and each cyst was crushed under a coverslip. The percentage of eggs parasitized by fungi was estimated under a microscope. The egg-parasitic index (EPI), a measure of the percentage of eggs, expressed on a scale of 0–10 corresponding to 0–100% of eggs, within a cyst that show microscopic evidence of invasion by fungal hyphae or spores (Chen et al., 1996), was used to estimate parasitism by fungi. The average EPI of a plot was the average of EPI from 50 cysts. Insufficient cysts were obtained for the S1 midseason 2015 sample and this sample was omitted from analysis.

### Cyst DNA extraction and metabarcoding

The surface sterilized cysts were crushed using a small pestle in a 1.5-ml microfuge tube, and DNA was isolated from the mixture using a modified CTAB method as described in Hu et al. (2017). The PCR amplification, library preparation and sequencing were carried out at the University of Minnesota Genomic Center, Saint Paul, MN USA. The universal fungal primers targeting the fungal internal transcribed spacer 1 (ITS1) barcode region that were used for this study included ITS1F (CTTGGTCATTTAGAGGAAG^*^TAA) and ITS2R (GCTGCGTTCTTCATCGA^*^TGC). The Illumina index and flow cell adapters were added together with the ITS1 primer sequences during the first PCR step, and then forward and reverse barcode sequences were added during the second PCR step using a dual-index PCR approach (Gohl et al., 2016). The cycling condition for first step PCR was: 95°C for 5 min; and then 25 cycles of 98°C 20 s, 55°C 15 s, and 72°C 1 min; followed by 72°C for 5 min as the last step. The second step PCR cycling was: 95°C for 5 min; and then 10 cycles of 98°C 20 s, 55°C 15 s, and 72°C 1 min; followed by 72°C for 5 min as the last step. The samples from the same year were pooled and sequenced on one Illumina MiSeq PE lane. The 2015 samples were sequenced with the 2 × 250 bp kit, and the 2016 samples were sequenced with 2 × 300 bp kit. For each MiSeq lane, control samples were included in order to assess the PCR and sequencing error in the downstream pipeline. The control samples included PCR negative control samples (with water added instead of DNA), samples of a mock community which was constructed by mixing equal quantities of DNA isolated from pure cultures or herbarium specimens from 36 taxa from across the fungal kingdom and nine randomly chosen cyst samples for technical replicates, for which libraries were independently prepared and amplified by PCR.

### Sequence quality control and processing

Together, the platform yielded a total of 39,282,202 reads for the two combined runs. Mothur v.1.35.1 (Schloss et al., 2009) was used to pair the sequences and make contigs, and those sequences that did not have more than 100 bp overlap were excluded. The remaining sequences that had <2 bp mismatch to the fungal ITS1 region primer were kept for further filtering. The last filtering step in mothur removed those sequences that had more than 8 homopolymers, had any ambiguous nucleotides, and/or were shorter than 200 bp or longer than 400 bp. After filtering, there was an average of 88,909 reads per sample, with the highest 209,442 and the lowest 9,541 per sample. One sample was excluded from the downstream analysis because of extremely low read count. The high quality filtered sequences from mothur were then transferred into QIIME v.1.9.1 (Caporaso et al., 2010). Chimeras were detected in QIIME using the UCHIME v.4.2.40 (Edgar et al., 2011) pipeline, and operational taxonomic units (OTU) (Blaxter et al., 2005) were picked using a *de novo* OTU picking approach using 97% similarity as a clustering threshold. Taxonomy was assigned by blasting the representative sequence of each OTU against the UNITE database (version 7.2, 06-28-2017) (Kõljalg et al., 2013). OTUs that had <10 sequence reads across samples were removed prior to downstream statistical analyses. We recovered 21 fungal species out of 36 for the mock community. The negative control samples had low OTU counts (<100), and there was no difference in terms of OTU counts between PCR technical replicate samples. After removal of poor quality reads and chimeras, the rarefaction curve of the fungal communities showed that the average sequencing depth of 50,000 per sample most likely captured most of the diversity of the fungal community (Figure S1) as the slope of the number of OTUs leveled off without much further increase after 50,000 reads.

### Statistical analysis

#### SCN egg density and EPI

Analysis of variance was performed in R v.3.2.3 (R Core Team, 2016). The homogeneity of variance of the SCN egg density was tested before fitting the data into a linear model and since variances were homogenous no transformation was needed. The effects of crop sequences and season were analyzed within each year. The crop sequence and season were fitted into a linear model as main effects with four replicates with the interaction between the two included in the model (Y ~ Replicate + CropSequence + Season + CropSequence:Season). Two way ANOVA was used to test the significance of the effects and this model was used for all the variables that were fitted into a linear model. A boxplot of SCN egg density by crop sequences was generated using “ggplot2” (Wickham, 2009). The same linear model and analysis pipeline was applied to the EPI data. Tukey's HSD tests were used to compare different treatment means between crop sequences or seasons. Significant effects were reported at *P* < 0.05 level through all the results unless otherwise stated.

#### Fungal community diversity

The OTU tables generated by QIIME were transferred to R for further analyses. All the relative abundance of phylum, class and genus was transformed with log_2_ (*x*+1). For all relative abundance analyses, the *P*-value was adjusted using the FDR method (Benjamini and Hochberg, 1995). Rarefaction curves were generated using the “rarecurve” function in the package “Vegan.” Alpha diversity indices (Chao1 and Shannon) were calculated using the functions in packages “Phyloseq” (McMurdie and Holmes, 2013). The same ANOVA model was used as above to compare the significance of Chao1 and Shannon index across season and crop sequences. For beta diversity, the OTU counts were transformed to relative abundance of each sample, and normalized by using a log_2_ (*x*+1) transformation. A Bray–Curtis dissimilarity matrix was calculated using the R package “Vegan” (Oksanen et al., 2011), and the significance of dissimilarity between treatments was tested using the Adonis function in Vegan. The mean distance between four replicates and centroid of each sample was calculated using “betadisper” to show the dispersion of the community, and Tukey's HSD was used to test the differences of the dispersion parameter between crop sequences or seasons. The function “procrustes” in package “Vegan” was used to detect fungal community similarity between different seasons and crop sequences across seasons within each year. The figures were generated with package “ggplot2” (Wickham, 2009).

Fungal taxa were assigned to a functional ecological guild using FUNGuild v.1.1 (Nguyen et al., 2016), which was used to construct a guild community matrix. The effects of season and crop sequence were analyzed with the same ANOVA model as above. We also created guilds consisting of the three main ecological groups of known nematophagous fungi (1) nematode-trapping, (2) egg-parasites, and (3) J2 endoparasites. Taxa were assigned to each group based on published reports in the literature demonstrating their isolation directly from SCN eggs (egg-parasites), ability to colonize J2s (endoparasites), or evidence of formation of trapping structures (trapping fungi) (Table S1). The relative abundance of all fungi belonging to a guild was summed, and the same ANOVA model was used to detect the differences of the egg-parasitic fungi across season and crop sequences. Again, the relative abundance of the potential egg-parasitic fungi was transformed using log_2_ (*x*+1).

## Data accession

The raw sequences were deposited into the NCBI database (Accession number: PRJNA415468).

## Results

### Effect of crop sequences on SCN egg density and EPI

**Figure 2:**
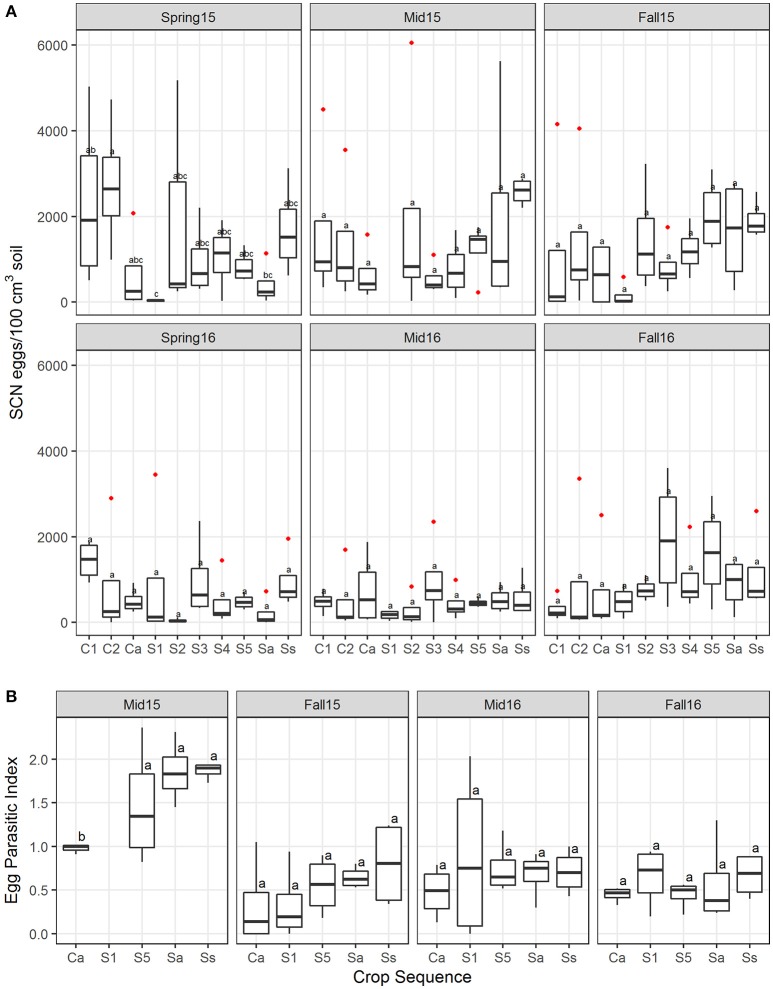
Soybean cyst nematode egg population density and EPI. **(A)** Boxplots showing the median and interquartile ranges across four replicates of SCN egg density measured as the number of eggs/100 cm^3^ soil at each sampling season (spring, midseason, and fall in 2015 and 2016). **(B)** Egg-parasitic index (EPI) across crop sequences and season showing median and first through fourth quartiles. Letters indicate differences that were significant (*P* < 0.05) by Tukey's LSD test and red dots are outliers. Crop sequence treatments include first (C1) and second (C2) year of corn following a 5 year soybean rotation, annual rotation of corn (Ca) following annual rotation of soybean (Sa), years one through five (S1–S5) of soybean following a 5 year corn rotation, and continuous corn (Cc) and susceptible soybean (Ss) monoculture.

Analyses using linear modeling showed that crop sequence had a significant effect on the SCN egg density across all crop sequence treatments in 2015 but not 2016 (Table S2). Overall in 2015, the first year of corn (C1) following a 5 year soybean rotation had the highest median egg population density. However, in midseason and fall, the continuous soybean monoculture (Ss) treatment had the highest density. In both years, early years of soybean (S1–S2) following a 5 year corn rotation generally had lower SCN egg population densities than other treatments (Figure 2A). The data also showed a trend of increasing SCN egg density with increasing years of soybean monoculture in midseason of 2015 and fall of both 2015 and 2016, but a significant correlation between increasing SCN egg density and increasing soybean year was only observed in fall of 2015 (Y ~ 86.6 + 350.95 ^*^Soy Year, *R*^2^ = 0.24, *P* = 0.02).

The estimates of EPI collected from each cropping treatment in both midseason and fall are an independent estimate of abundance of fungal egg parasites that measures the percentage of eggs in cysts colonized by fungi. Cysts in soybean plots had a slightly higher average EPI than corn plots at midseason and fall of both years (Figure 2B), although this difference was only significant in midseason of 2015. There was also a trend of increasing EPI with increasing years of soybean (Figure 2B), but no significant correlation with year of soybean was observed (Table S3). In analyzing correlations between egg density and EPI, a significant positive correlation with egg density was observed only in fall of 2015 (Table S3).

### Fungal taxa found in cysts

**Figure 3:**
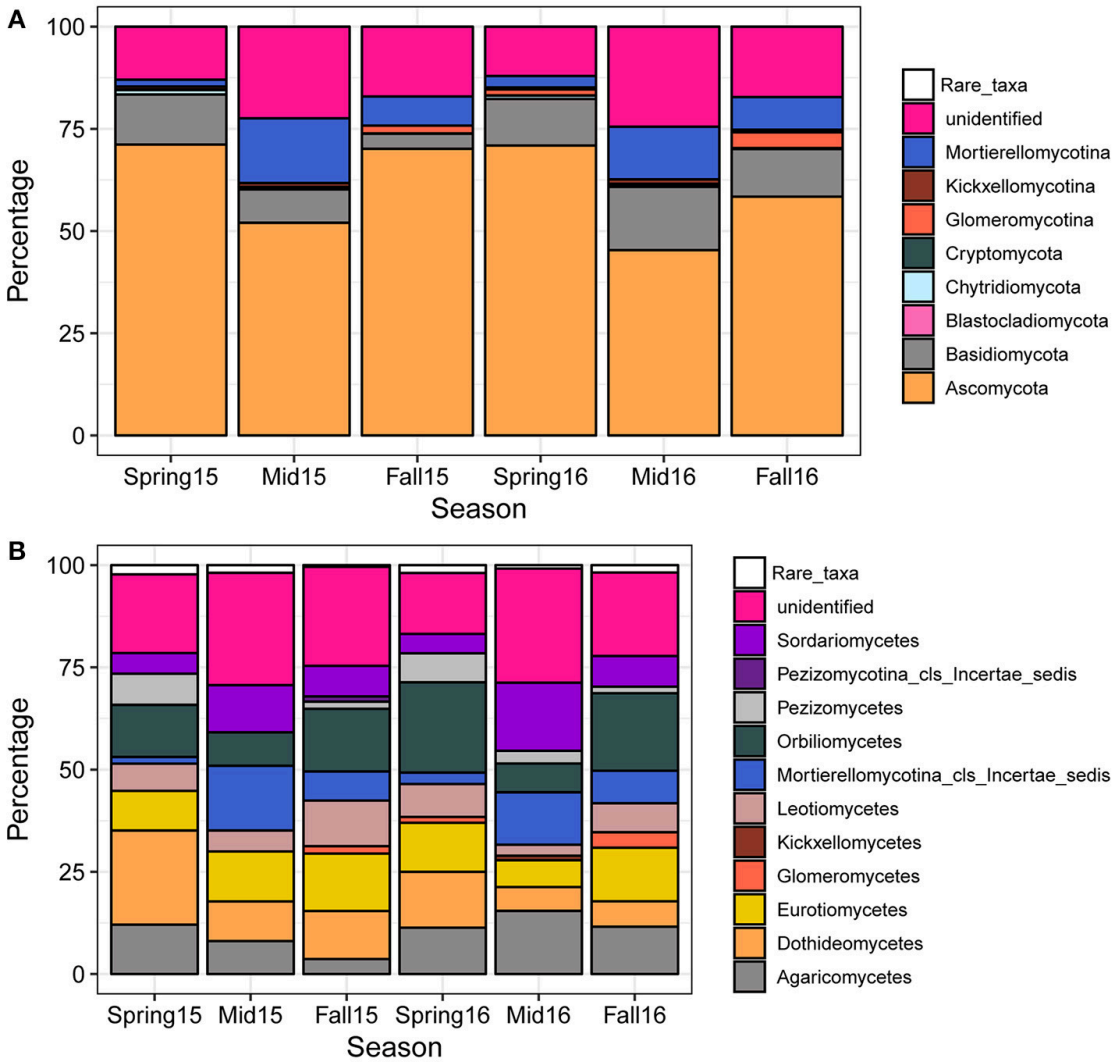
Percentage of fungal OTUs mapping to fungal phyla, subphyla, and classes across seasons. Each bar represents the average proportion of phyla **(A)** or classes **(B)** of all the samples within the sampling time point (spring, midseason, and fall). The category “Rare_taxa” includes all classes that had <0.1% relative abundance.

A total of 1964 fungal OTUs were identified (Table S4), with the OTU count in a single sample averaging close to 150 (Figure S1). The percentage of OTUs mapping at the phylum and subphylum levels consisted of predominantly Ascomycota, followed by Basidiomycota, Mortierellomycotina, and Glomeromycotina, although a relatively large proportion (approximately 20%) of OTUs could not be identified to fungal phyla or subphyla (Figure 3A). More rare phyla and subphyla with <1% of total OTUs included Kickxellomycotina, Chytridiomycota, Cryptomycota, as well as other early diverging fungal phyla and subphyla that contained on average <0.01% of total OTUs (Blastocladiomycota and other rare taxa) (Figure 3A). In all seasons, Ascomycota predominated, comprising more than half of the OTUs at each sampling time point (Figure 3A). Analysis of variance across the growing season showed that Ascomycota were generally decreased at midseason, while Basidiomycota and Chytridiomycota showed a higher percentage of taxa in spring than in midseason and fall in 2015 (Figure 3A; Table S5). In both years, Mortierellomycotina was significantly increased at midseason and fall compared to spring, while Glomeromycotina was significantly increased in fall compared to spring and midseason (Figure 3A; Table S5).

At the class level, the highest number of OTUs fell within the Orbiliomycetes, Dothideomycetes, Eurotiomycetes, Sordariomycetes, Leotiomycetes, and Pezizomycetes in Ascomycota, the Agaricomycetes in Basidiomycota, the Glomeromycetes in Glomeromycotina, and the Mortierellomycotina_cls_Incertae_sedis in Mortierellomycotina (Figure 3B). Patterns of relative abundance observed at the subphyla level for Mortierellomycotina and Glomeromycotina were consistent at the class level. Mortierellomycotina_cls_Incertae_sedis were significantly increased at midseason and fall compared to spring in both years, while Glomeromycetes, which include the arbuscular vesicular mycorrhizal fungi (AMF), were significantly increased in fall during both years (Figure 3B; Table S5). In contrast, Orbiliomycetes, Dothideomycetes, and Leotiomycetes showed the opposite pattern with a decrease at midseason, but this trend was only significant in 2016 (Figure 3B; Table S5). A few individual genera also changed significantly across season, with *Mortierella* and *Nectria* increasing at midseason in 2015 and 2016, respectively, *Leptosphaeria* increasing in spring of 2015, and *Exophiala* increasing in fall of 2016 (Table S5).

### Fungal community alpha diversity

The Chao1 index showed that the average number of OTUs in the fall was greater than in spring and midseason in 2016 but not 2015 (Table 2). The crop sequence treatment had a significant effect on alpha diversity only in spring and fall of 2016 (Table 3). In spring of 2016, annual rotation with corn (Ca) as well as longer soybean rotations (S3 and S5) had significantly greater total number of OTUs than S1. In fall 2016, however, the first year of rotation after a 5 year sequence (C1, S1), as well as several soybean treatments (S2, S5, Sa) had significantly higher numbers of OTUs based on the Chao1 richness index than corn (Ca, C2) (Table 3). The Shannon index, another alpha diversity index which takes into account the evenness of the community, yielded results mostly consistent with the Chao1 richness index (Table 3).

**Table 2:** Fungal community alpha diversity indexes across season.

**Season**	**2015**		**2016**	
	**Chao1**	**Shannon**	**Chao1**	**Shannon**
Spring	136.0a	2.21b	141.9b	2.33b
Mid	140.4a	2.26b	141.6b	2.16b
Fall	150.9a	2.44a	163.9a	2.62a
**ANOVA**				
CropSeq	0.4	0.039^*^	0.002^**^	0.015^*^
Season	0.24	0.017^*^	0.003^**^	<0.001^***^
CropSeq^*^Season	0.97	0.38	0.03^*^	0.03^*^

The data are means across all 40 samples, 10 crop sequences each with four replicates within each season. Different lowercase letters indicate a significant difference across seasons at P < 0.05 by Tukey's HSD test. Significant differences by ANOVA are reported at

* P < 0.05,

** P < 0.01, and

*** *P < 0.001*.

**Table 3:** Fungal community alpha diversity indexes across crop sequences.

**Cropseq**	**2015Spring**		**2015Midseason**		**2015Fall**		**2016Spring**		**2016Midseason**		**2016Fall**	
	**Chao1**	**Shannon**	**Chao1**	**Shannon**	**Chao1**	**Shannon**	**Chao1**	**Shannon**	**Chao1**	**Shannon**	**Chao1**	**Shannon**
C1	104a	2.11a	188a	2.27a	123a	1.80a	163ab	2.67ab	129a	2.27a	195a	2.73a
C2	114a	2.06a	135a	2.19a	98a	2.11a	111ab	1.85ab	141a	2.42a	133b	1.83b
Ca	125a	1.80a	111a	2.66a	83a	2.18a	179a	2.66a	140a	2.10a	154ab	2.54ab
S1	156a	1.43a	NA	NA	62a	2.26a	97b	2.24b	115a	1.72a	199a	3.00a
S2	140a	1.73a	153a	2.13a	98a	2.10a	152ab	2.12ab	187a	2.29a	203a	2.74a
S3	141a	2.19a	122a	2.08a	138a	1.68a	181a	2.51a	161a	2.20a	164ab	2.41ab
S4	182a	2.20a	109a	2.13a	126a	2.35a	169ab	2.70ab	171a	2.25a	148a	2.68a
S5	138a	1.84a	169a	2.63a	72a	2.47a	173a	2.63a	188a	2.35a	203a	2.92a
Sa	168a	1.96a	119a	2.05a	109a	2.57a	135ab	2.00ab	145a	2.25a	184a	2.80a
Ss	154a	1.91a	149a	2.45a	93a	2.42a	131ab	1.94ab	129a	1.78a	163ab	2.57ab
*P-value*	0.73	0.56	0.64	0.27	0.53	0.51	0.01^*^	0.03^*^	0.16	0.40	0.04^*^	0.01^*^

Data were means of four replicates. Different lowercase letters indicate a significant difference across crop sequences at P < 0.05 by Tukey's HSD test. P-value was adjusted with FDR and significance level set at corrected

* *P = 0.05*.

### Fungal community composition over seasons and crop rotation sequences

**Figure 4:**
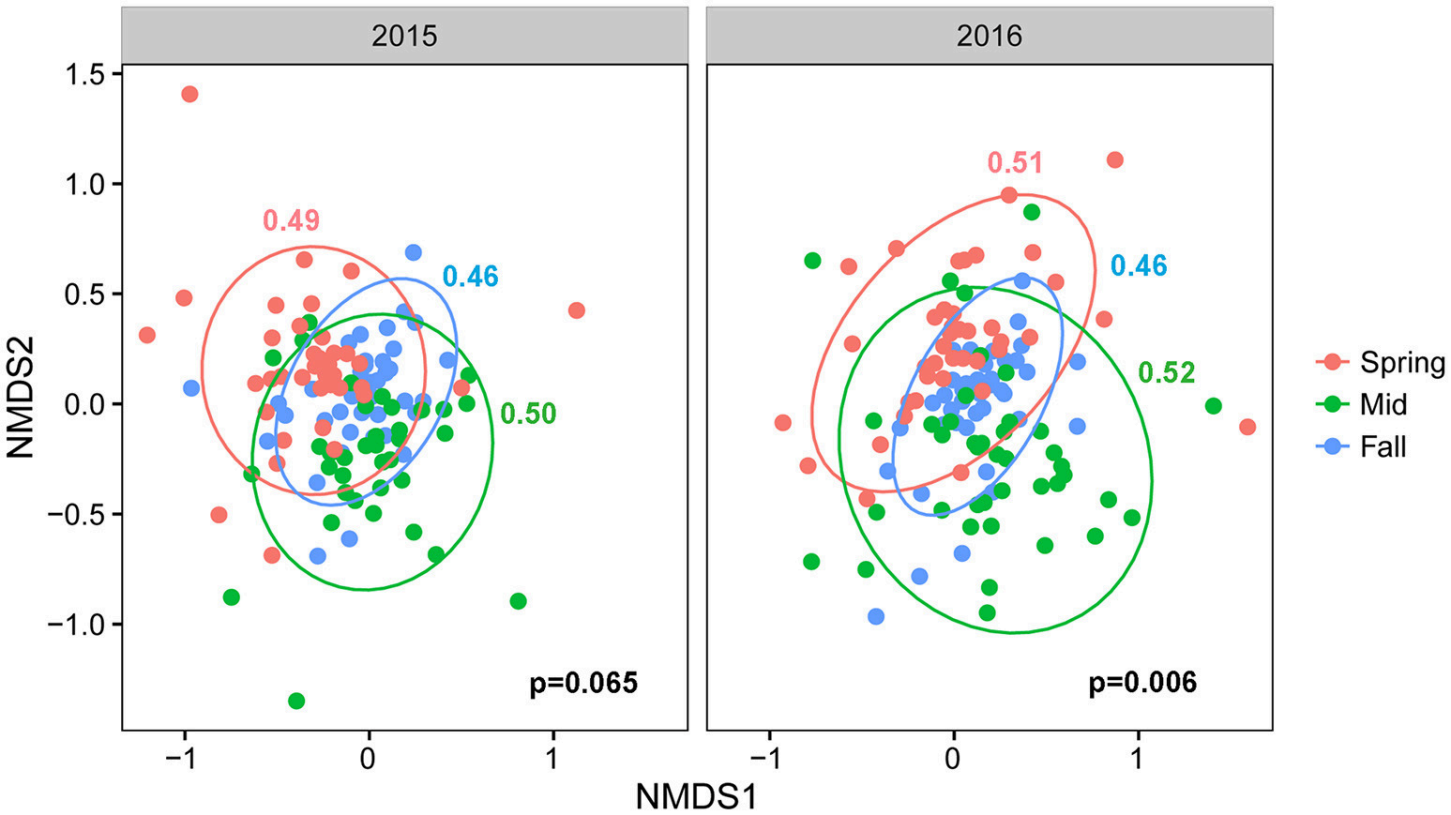
NMDS plot based on Bray–Curtis distance between fungal communities by season (pink = spring, green = midseason, and blue = fall). Ellipses are drawn at 95% confidence limit, *P*-value for ANOVA comparing beta-dispersion values are shown on bottom right of graph and dispersion parameters for each season color coded according to season and shown next to the corresponding ellipse.

Procrustes analyses comparing overall community taxonomic composition for all treatments combined across sampling time points showed that fungal communities in spring, midseason, and fall differed significantly from each other in both years (Table S6). An NMDS plot of all treatments combined across seasons in 2015 and 2016 showed that the communities from different crop sequence treatments clustered together more closely in fall with a lower beta-dispersion parameter, a measure of how similar communities are to one another, indicating that the communities were the most similar in fall (Figure 4). The communities at midseason had a higher beta-dispersion parameter and were thus more dissimilar from one another (Figure 4). Procrustes tests, however, showed that the fungal community structure of each individual crop sequence treatment was relatively stable over time across a season, with only S4 in 2015 and Ss in 2016 being significantly different from spring to midseason (Table S6).

**Figure 5:**
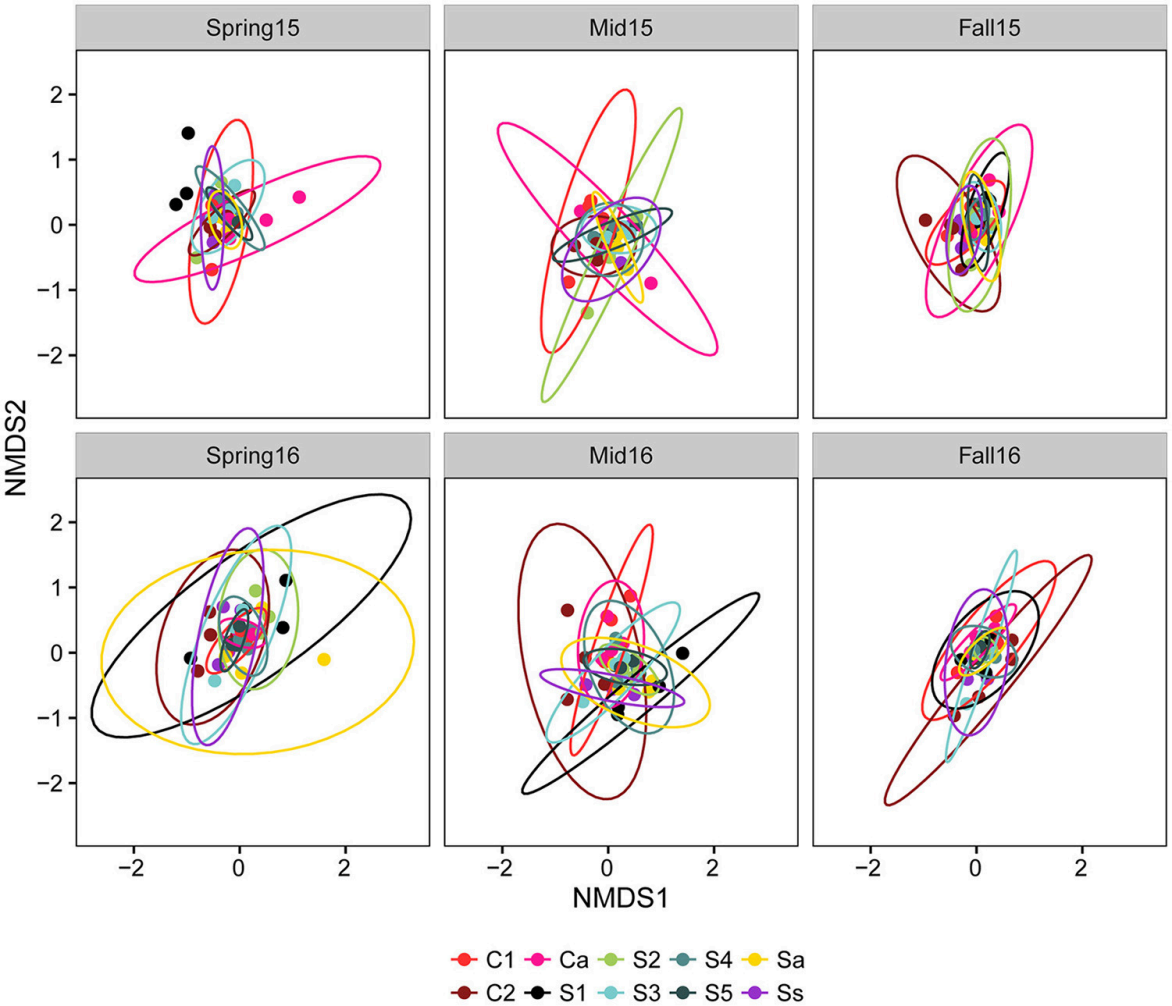
NMDS plot based on Bray–Curtis distance between fungal communities by crop sequences. Crop sequence treatments include first (C1) and second (C2) year of corn following 5 year soybean rotation, annual rotation of corn (Ca) following annual rotation of soybean (Sa), years one through five (S1–S5) of soybean following a 5 year corn rotation, and continuous corn (Cc) and susceptible soybean (Ss) monoculture. Ellipses are drawn at 95% confidence limit.

The Adonis test using the Bray–Curtis distance matrix suggested significant interactions between season and crop sequence treatment (Table 4). Thus, the crop sequence effect was analyzed separately within each season. Overall, the community composition was significantly different across crop sequences at all sampling time points (Table 4). When comparing all soybean treatments to all corn treatments, the fungal community in the cysts was significantly different in fall and midseason but not spring in both years. There were significant differences among soybean crop sequence treatments at all sample time points except midseason of 2015 (Table 4). NMDS plots and comparisons of beta-dispersion parameters found that dispersion parameters were significantly different across all crop sequence treatments. Communities in the cysts from increasing years of soybean (e.g., S1–S5) and continuous soybean monoculture generally had lower dispersion parameters and were thus more similar to one another than soybean rotated with corn or continuous monoculture (Figure 5; Table S7).

**Table 4:** *P*-value of Adonis test of Bray–Curtis dissimilarity matrix across crop sequence treatments within each season.

**Treatment^a^**	**Spring15**	**Mid15**	**Fall15**	**Spring16**	**Mid16**	**Fall16**
Allcrop sequences	0.002^**^	0.006^**^	<0.001^***^	<0.001^***^	<0.001^***^	<0.001^***^
Soy vs. Corn	0.94	0.001^**^	<0.001^***^	0.17	<0.001^***^	<0.001^***^
Soy only	0.002^**^	0.28	0.014^*^	<0.001^***^	0.002^**^	0.003^**^

a All crop sequences, included comparison across all 10 crop sequences; Soy vs. Corn, compared between the all treatments from each of two different crops; soy only, only compared among sequences which contained soybean grown in the year. Significant differences are reported at FDR corrected

* P < 0.05,

** P < 0.01, and

*** *P < 0.001*.

### Fungal taxa affected by crop sequences

**Figure 6:**
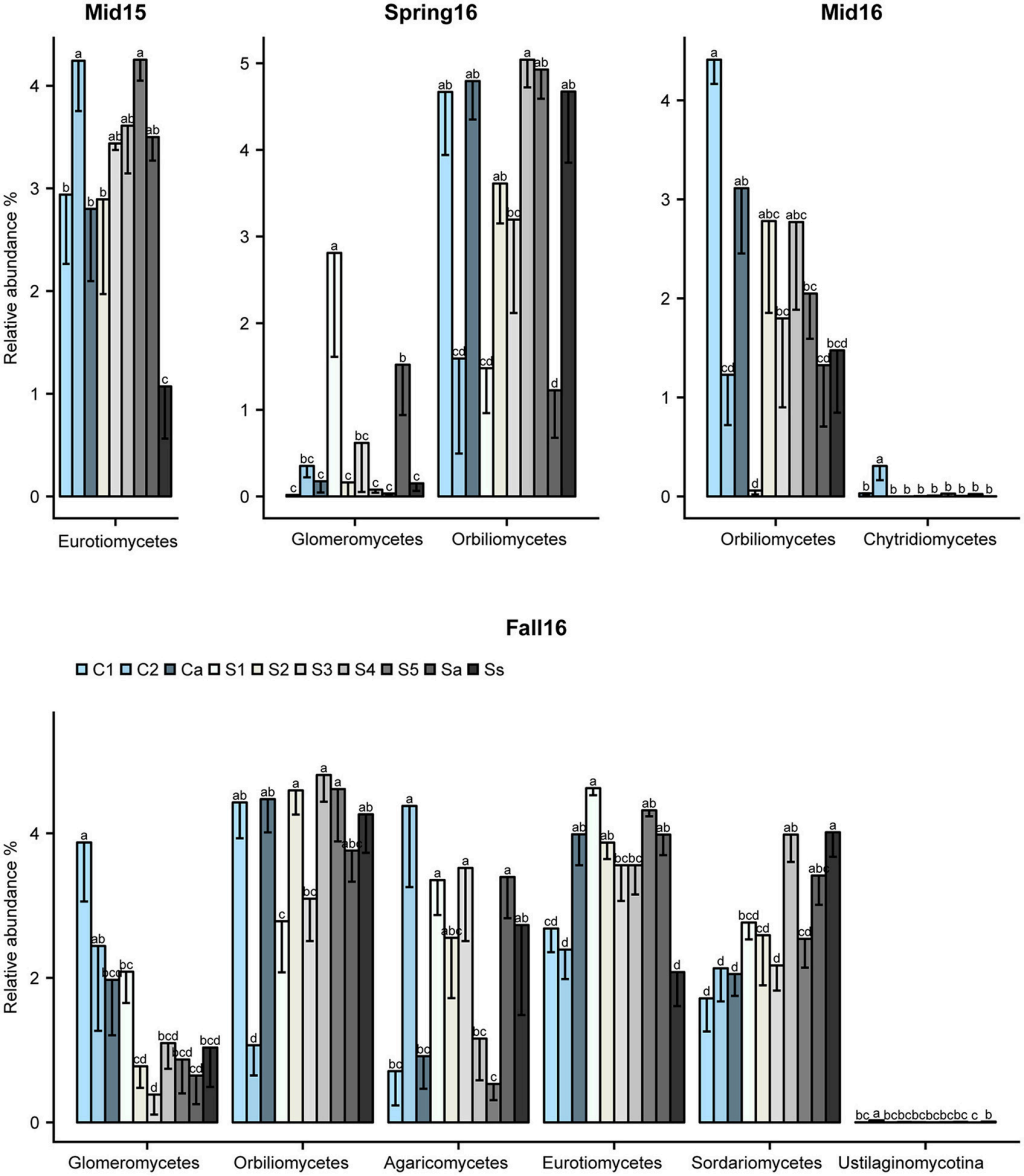
Fungal classes affected by crop sequences. Relative abundance of classes of fungi that differed significantly by ANOVA across crop sequence treatments. Crop sequences include first (C1) and second (C2) year of corn following 5 year soybean rotation, annual rotation of corn (Ca) following annual rotation of soybean (Sa), years one through five (S1–S5) of soybean following a 5 year corn rotation, and continuous corn (Cc) and susceptible soybean (Ss) monoculture. Different letters indicate significant (*P* < 0.05) differences in relative abundance detected by Tukey's HSD test.

The crop sequence effect on fungal taxa varied with sampling time point. At the class level, Eurotiomycetes differed significantly across crop sequence treatments in midseason of 2015 and fall of 2016, and were generally more abundant in soybean treatments compared to corn (Figure 6). However, they were highly abundant in C2 at midseason 2015 and annual corn following soybean (Ca) in fall 2016 (Figure 6). The class Orbiliomycetes, which includes most of the genera of nematode-trapping fungi, was significantly affected by crop sequence in spring, midseason, and fall of 2016. In addition, they had higher average relative abundance in longer term soybean (S4-5 and Ss), as well as alternating soybean and corn (Sa, Ca) and the first year of corn following 5 years of soybean (C1) in both spring and fall of 2016 (Figure 6). They had significantly reduced abundance in C2, the second year of corn (Figure 6). The Glomeromycetes had significantly higher relative abundance in soybean following 5 years of corn (S1) in spring of 2016. However, in fall of 2016, early corn treatments (C1-2) showed higher relative abundance of Glomeromycetes than all other crop sequences (Figure 6). Other classes that showed significant effects of crop treatment in fall of 2016 included Sordariomycetes, Eurotiomycetes, Agaricomycetes, and Ustilaginomycotina_cls_Incertae_sedis (Figure 6). Sordariomycetes, which include the order Hypocreales that contains many egg-parasites and endoparasites of nematodes (Sung et al., 2007), had higher relative abundance in longer term soybean plots (S5 and Ss) (Figure 6).

**Figure 7:**
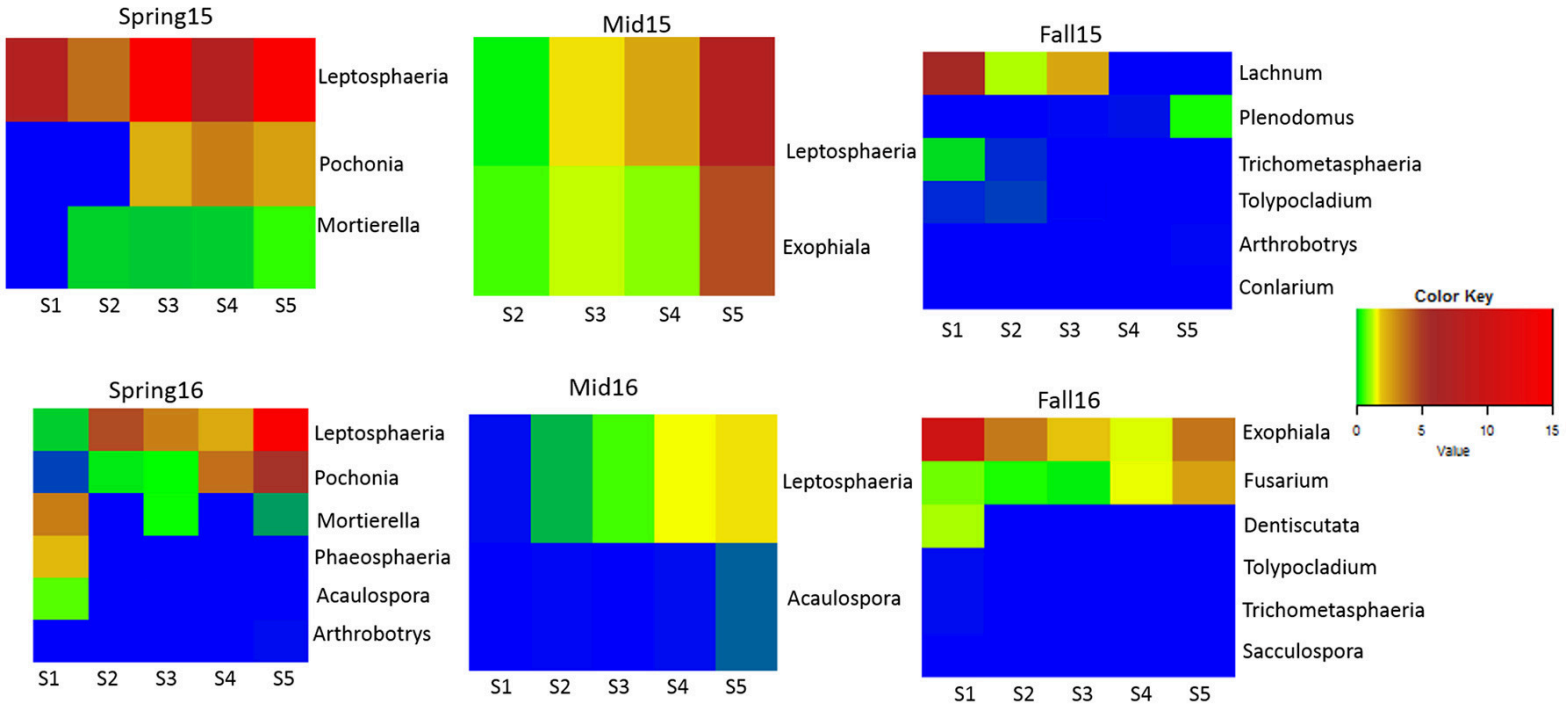
Heatmap of fungal genera significantly correlated with years of soybean monoculture. Relative abundance is color coded from lowest (blue: 0–0.1%) to highest (red: 5.1–15%) across soybean crop sequence treatments S1–S5.

Only a few genera differed significantly across all crop sequence treatments by ANOVA. In fall of 2015, *Phacidium* had significantly greater abundance in the second year of corn (C2) (Table S7). In fall 2016, *Exophiala* showed greatest abundance in the S1 treatment, followed by Sa, and showed lowest abundance in the C1, C2 treatments as well as continuous soybean monoculture (Ss) (Table S8). However, a number of genera were found to be significantly correlated, either positively or negatively, with increasing years of continuous soybean monoculture. The genus *Leptosphaeria* showed the most consistent trend, showing a significant positive correlation with longer term soybean treatments in midseason of 2015 and spring and midseason of 2016 (Figure 7; Table S9). The relative abundance of *Exophiala* and *Fusarium* also increased significantly with increasing years of soybean in midseason of 2015 and fall of 2016, respectively (Figure 7; Table S9). The relative abundance of both *Mortierella* and *Pochonia* (Syn. *Metacordyceps*) increased in longer term soybean in spring of both years (Figure 7; Table S9). In contrast, several taxa (*Arthrobotrys, Aculospora, Trichometasphaeria*, and *Tolypocladium*) showed a negative correlation with increasing soybean year in either spring and/or fall of each year (Figure 7; Table S9).

### Fungal taxa correlated with SCN egg density and EPI

Several fungal taxa were significantly correlated with SCN egg density in spring and midseason but not fall in both years. The taxa most significantly and positively correlated with egg density included *Harpellomyces montanus* in Kickxellomycotina in both spring and midseason of 2015 and the Dothideomycete *Neophaeosphaeria agaves* in spring 2015, midseason 2015, and spring 2016, and *Mortierella alpina* and an unidentified *Eurotiales* species in spring of 2016 (Table S10). One other species of *Mortierella, Mortierella elongata*, was also positively correlated with egg density in spring 2016, along with several Ascomycota (*Parastagonospora, Alternaria*, and *Trichoderma parapiluliferum)*, Basidiomycota (*Phallus* and *Microstromatales*), and Chytridiomycota (*Rhizophlyctis*) (Table S10). Only a few other taxa were positively correlated with egg density in midseason, including unidentified *Sarcosomataceae* and *Phanerochaete* species and *Diaporthe phaseolorum* in 2015, and *Hannaella oryzae* and an unidentified *Eurotiales* taxon in 2016 (Table S10).

Eight fungal taxa were found to be significantly correlated with EPI in midseason of 2016. Those taxa including several ascomycete genera (*Tubeufia, Geosmithia, Cylindrocarpon*, and *Nectria*) as well as basidiomycetes (an unidentified species in family Bolbitiaceae and an unidentified species in order Agaricales) and taxa from several early diverging fungal subphyla (unidentified phyla Blastocladiomycota, and Chytridiomycota) (Table S10). There was no significant correlation between fungal taxa and EPI in midseason or fall 2015, or in fall 2016.

### Fungal ecological guilds found in SCN cysts

**Figure 8:**
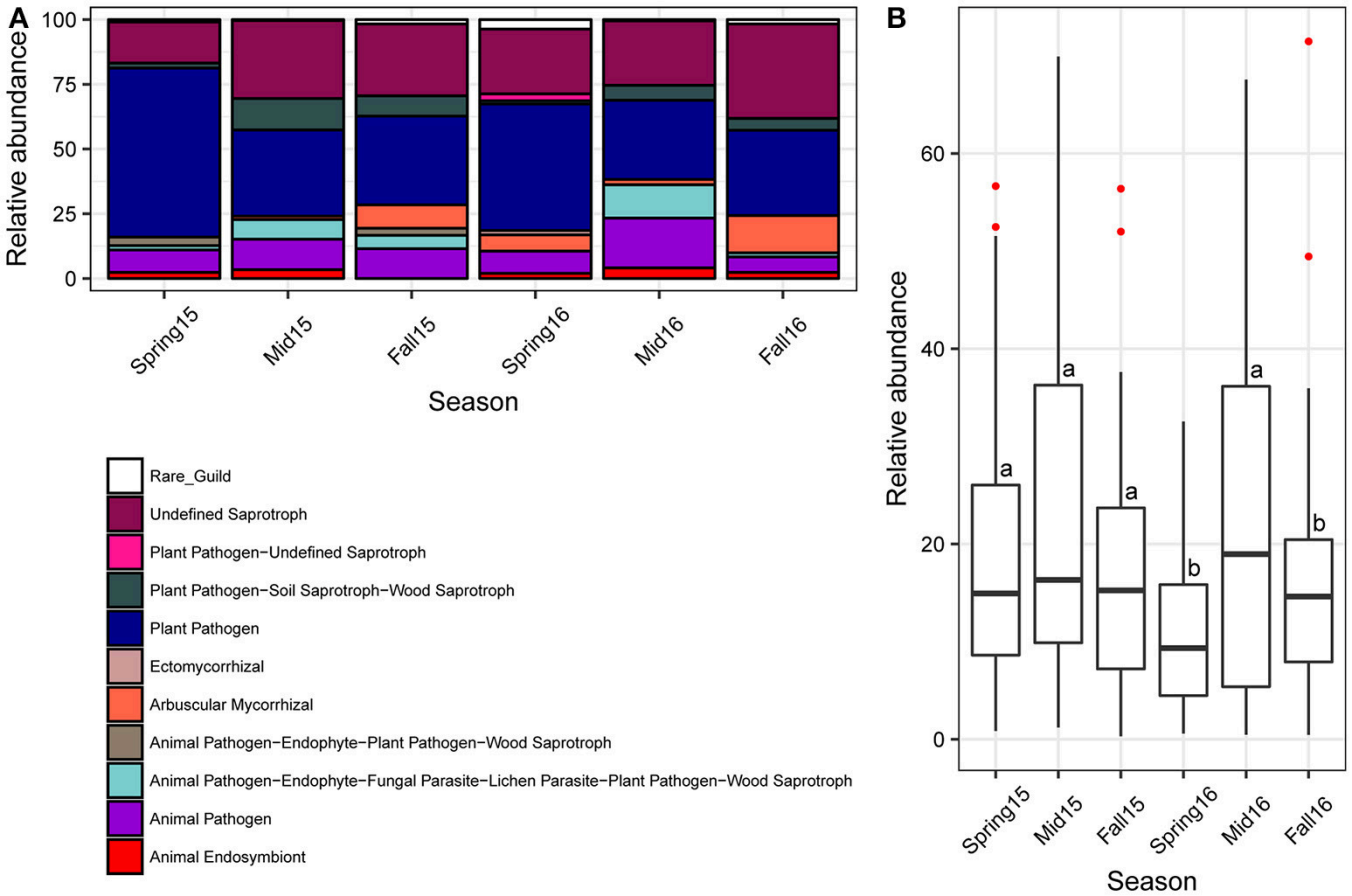
Fungal ecological guilds across season. **(A)** Relative abundance of FUNGuild categories found in cysts, color coded according to legend. The category “Rare_Guild” refers to guilds that had <0.1% relative abundance. **(B)** Barplots of relative abundance of potential nematode egg-parasitic fungi identified through literature searches across season showing median and first through fourth interquartile ranges with red dots indicating outliers. Different letters indicate significant differences at *P* < 0.05 across crop sequences using Tukey's HSD test. Red dots indicate outliers.

Based on the v.1.1 release of the FUNGuild database, the guilds of Plant Pathogen and Undefined Saprotroph had the largest percentages of assigned OTUs, while other moderately abundant guilds included Ectomycorrhizal, Animal Pathogen, Animal Pathogen-Endophyte-Plant Pathogen, Plant Pathogen–Soil Saprotroph–Wood Saprotroph, and Arbuscular Mycorrhizal (e.g., AMF) (Figure 8A; Table S11). All the remaining guilds comprised <2.5% of OTUs. However, a large portion of OTUs could not be assigned to an ecological guild due to lack of published data on the lifestyle and ecology of many fungi.

When considering only high confidence rank guilds and excluding unassigned OTUs, several guilds differed significantly across seasons (Figure 8A; Table S12). Arbuscular Mycorrhizal were highly significantly increased in fall of 2015, while Plant Pathogens were highly significantly decreased at midseason and fall of 2015. Plant Pathogen-Soil Saprotroph-Wood Saprotroph were significantly increased in midseason compared to spring and fall in both years, while Animal Pathogen-Endophyte-Fungal Parasite-Lichen Parasite-Plant Pathogen-Wood Saprotroph were significantly increased in midseason compare to fall in both years (Figure 8A; Table S12). The Animal Pathogen-Endophyte-Fungal Parasite-Lichen Parasite-Plant Pathogen-Wood Saprotroph guild included taxa within the family Nectriaceae in Hypocreales, which have been previously isolated from SCN cysts (Tables S1, S8) (Carris et al., 1989; Chen and Chen, 2002). Although the Animal Pathogen guild alone was not significantly affected by crop sequences, it also showed a trend of increased relative abundance at midseason in both years (Figure 8A).

### Fungal ecological guilds correlated with crop sequence, egg-density, and EPI

The crop sequence did not have much effect at the guild level except that a greater proportion of OTUs belonging to Arbuscular Mycorrhizal were observed in the corn treatments compared to the soybean treatments in fall of both 2015 and 2016 (Figure S2). There was no significant correlation between guild and year of soybean crop sequence in either year. However, several guilds were strongly correlated with egg-density in 2015. Animal Endosymbionts were positively correlated with egg density in both spring and midseason of 2015, and Plant Pathogens were strongly correlated with egg density in spring 2015 (Table S13). Several Plant Saprotrophic guilds were correlated with egg density in midseason 2015 (Orchid Mycorrhizal-Plant Pathogen-Wood Saprotroph and Wood Saptrotroph) and in Spring 2016 (Leaf Saptrotroph) (Table S13). Only one guild (Dung Saprotroph-Plant Saprotroph-Soil Saprotroph) was significantly correlated with EPI in midseason 2016 (Table S13).

### Candidate egg-parasitic fungi affected by season and crop sequences

**Figure 9:**
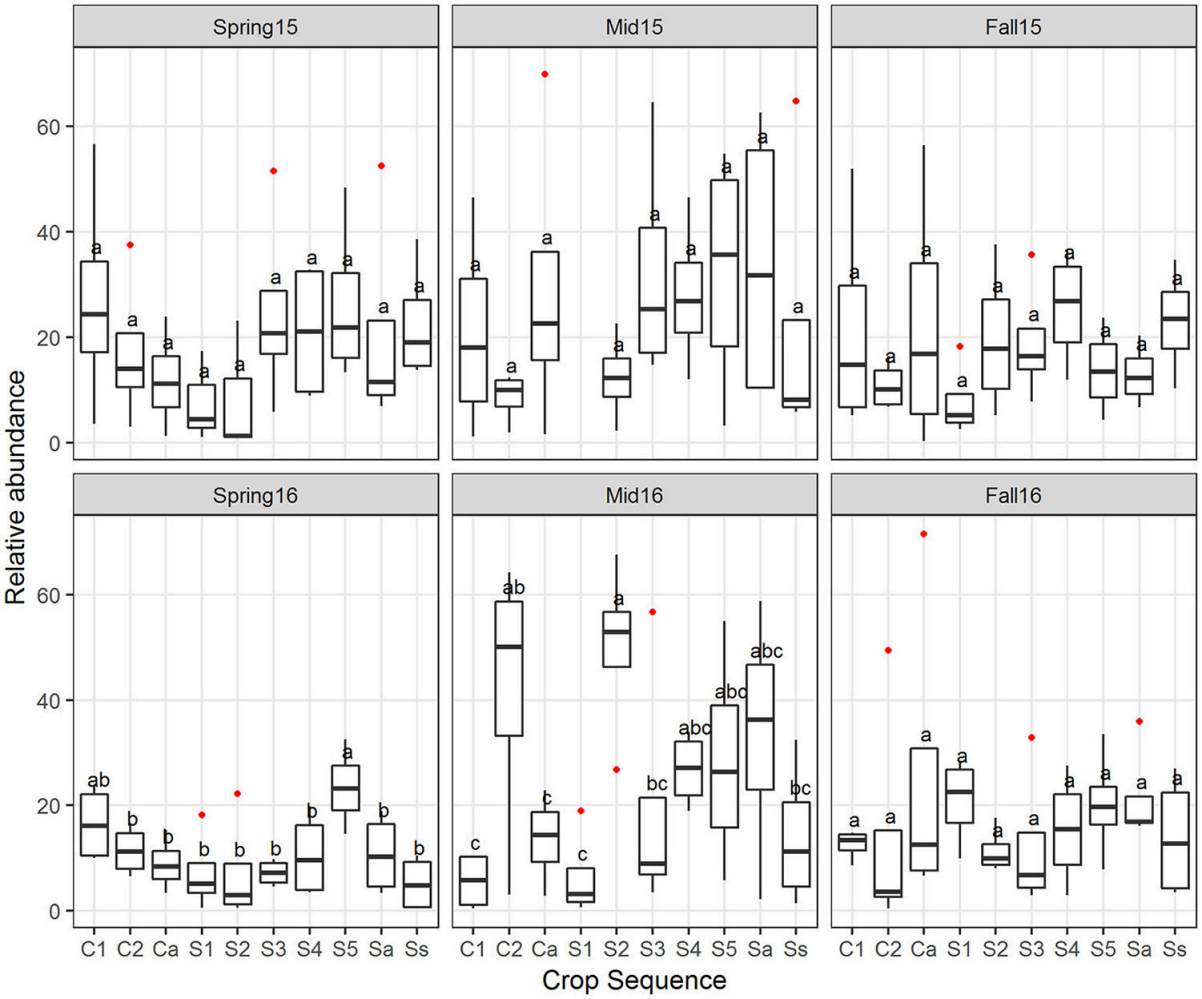
The relative abundance of potential egg-parasitic fungi affected by crop sequence. Boxplots showing median and interquartile range of relative abundance of potential egg-parasitic fungi across crop sequences. Crop sequences include first (C1) and second (C2) year of corn following 5 year soybean rotation, annual rotation of corn (Ca) following annual rotation of soybean (Sa), years one through five (S1–S5) of soybean following a 5 year corn rotation, and continuous corn (Cc) and susceptible soybean (Ss) monoculture. Different letters indicate significant differences at *P* < 0.05 across crop sequences using Tukey's HSD test. Red dots indicate outliers.

Classification of OTU into nematophagous guilds indicated that on average, about 25% of the OTUs belonged to nematode-parasitic fungi. Of all the nematode parasites detected, 98% were potential egg-parasitic fungi with evidence of previous isolation from SCN eggs in the literature (Table S1), 2% were trapping fungi, and no endoparasitic fungi were detected in the cysts. Overall, the potential egg-parasitic fungi had higher relative abundance in midseason than spring and fall in both years, but were significantly higher at midseason only in 2016 (Figure 8B). Across crop sequence treatments, the relative abundance of potential egg-parasitic fungi showed a decreasing trend from C1, C2, Ca to S1 in spring of both years (Figure 9). In contrast, the potential egg-parasitic fungi showed a trend of increasing median relative abundance with increasing soybean year at spring and midseason of both years (Figure 9). However, a significant correlation between relative abundance of egg-parasitic fungi and years of soybean was only detected in spring of 2016 (*y* = 0.43 + 3.6 (soybean year) with *r*^2^ = 0.27, *p* = 0.01). Significant differences across all crop treatments detected by ANOVA and Tukey's test were observed only in spring and midseason of 2016 (Figure 9). In spring of 2016, S5 had the highest relative abundance of potential egg-parasitic fungi, while continuous soybean monoculture (Ss) surprisingly had the lowest relative abundance of potential egg-parasitic fungi (Figure 9). In midseason of 2016, S2 had the highest relative abundance of potential egg-parasitic fungi, while C1 had the lowest proportion over all crop sequences (Figure 9).

## Discussion

In this study, the fungal communities in cysts of SCN under long term soybean-corn rotations showed seasonal variation during the growing season and were also affected by crop rotation treatments and their interaction. Our results show a strong seasonal effect on fungal communities inhabiting cysts. Although total numbers of species (Chao1) and diversity (Shannon Index) were the highest in the fall season (Table 2), the NMDS plots showed that communities appeared the most diverged from one another at midseason compared to spring and were the least divergent in the fall. In addition to an increase in taxonomic groups such as Mortierellomycotina, the percentage of OTUs mapping to potential egg-parasitic taxa as well as FUNGuild categories containing an Animal Pathogen lifestyle also increased at midseason of both years.

The fungal community in the cysts in relation to crop rotation has rarely been reported, but could provide valuable information for managing the SCN because crop rotation is the most common SCN management strategy employed in high soybean and corn-producing Midwestern states in the USA (Grabau and Chen, 2016b). In a previous study in an adjacent field, the soil in a long-term monoculture of SCN-susceptible soybean was suppressive to the SCN, potentially due to the accumulation of SCN parasites (Chen, 2007). Identification of fungal taxa correlated with increasing years of soybean monoculture, SCN egg-density, and EPI has potential to identify biocontrol organisms for the SCN. Similarly, understanding the effects of crop rotation on both nematode populations and the fungal communities in the cysts is important for optimizing crop rotation sequences to encourage establishment and maintenance of potential biocontrol organisms in agroecosystems. This study demonstrates that the number of years of continuous soybean clearly shapes the fungal community within the cysts and suggests that a 5-year rotation and even an annual rotation may be sufficient to reduce numbers of SCN parasites while also maintaining fungal antagonists of the SCN.

### Fungal community alpha diversity

The total number of species or OTUs observed remained fairly constant from spring to midseason, but increased from midseason to fall, indicating a potential influx of new species into the microbial community between midseason and soybean harvest. There are several possible explanations for this trend. One is that competition between fungi in the cysts is minimal and cysts simply accumulate a more diverse set of fungi over time as the reservoir of cyst-invading fungi in soil communities builds up over the growing season. Another is that some cysts collected in spring may have been produced in the previous season and were already colonized by fungi. Some data shows that “pre-colonization” of a cyst by parasitic or some saprobic fungi may establish priority effects through competition that prevents additional fungal species from invading the cysts (Chen and Chen, 2003). Nearly all of the cysts collected in spring and a much larger proportion of cysts collected at midseason than in fall were likely produced during the previous growing season. The life cycle of SCN is about 30 days under optimal conditions (Chen et al., 2001a) and the interval between spring and midseason sampling points was about 60 days, allowing the SCN to have completed one or perhaps two life cycles between these two sampling time points. Additionally, these newly produced young cysts may not have been colonized by many fungi, as a previous study showed that the frequency of fungi colonizing 30 day old cysts was low (Chen et al., 1994). In contrast, the time interval from midseason to fall was about 3 months, and it is likely that a larger proportion of cysts in fall are produced and colonized during the current growing season. The reservoir of species in the soil community may differ between years due to the effects of crop rotation on crop residues (Bending et al., 2002) and crop root exudates (Broeckling et al., 2008) as well as their effects on soil properties such as C:N ratio, organic matter content, and nutrient availability (Lauber et al., 2008). There was also a slight but not significant increase in the Chao1 index in the first year after either annual or 5 year soybean or corn rotations (e.g., S1, Sa, C1, and Ca), indicating that crop rotation may have some effect of increasing species richness of fungal communities in cysts (Table 3). A previous study on the impact of crop rotation on the nematode communities in this same field also showed that the largest shift in fungal communities occurred in the first year of corn or soybean after a 5 year rotation of monoculture of the alternate crop (Grabau and Chen, 2016b).

### Fungal community structure

The composition of fungal communities differed significantly from each other by season (spring, midseason, fall), indicating a dynamic change over the soybean and corn growing season. A procrustes analyses over sampling time points also suggested that the development of the fungal community in late season was not strongly influenced by “priority effects” and was not especially dependent on the fungal community colonizing cysts in the spring (Table S6). The fungal communities in the fall were more tightly clustered with lower dispersion parameters on the NMDS plot than those at midseason or spring, while communities had the highest dispersion parameter at midseason (Figure 5; Table S7). In soybean, cysts are formed on the roots and since they have limited mobility, they are likely to stay within close proximity to the roots when dislodged into soil if there is no physical disturbance. Thus, the rhizosphere communities developing over a season could also affect fungal communities in cysts. Since plant roots are most actively growing and secreting root exudates that support rhizosphere microbial communities during the midseason sampling point, the diversity of fungi in the rhizosphere available to colonize cysts may also peak at midseason.

Fungal communities inhabiting the local environment of the rhizosphere directly surrounding plant roots have been shown to be shaped by plant species and root exudates (Berg and Smalla, 2009; Berendsen et al., 2012). We show that crop rotation was also a significant factor affecting the composition of fungal communities in cysts. Fungal community β-diversity was influenced by crop rotation, with the dispersion parameter or dissimilarity between communities on NMDS plots generally decreasing over the first 5 years of monoculture treatments (S1–S5) (Figure 5; Table S7). Interestingly, however, the long term monoculture plots (Ss) generally had a higher dispersion parameter than S5, while the first year of soybean after corn (S1) had the highest dispersion parameters, especially in spring. Although differences were found across crop sequences, these effects could not be fully explained by only two axes. There are many additional factors that could have affected the development of fungal communities in cysts, such as soil physical and chemical characteristics (e.g., organic matter, nutrients, and soil moisture), the age of the cysts, and the population densities of SCN in the field.

### Fungal taxa affected by crop-rotation, season, SCN egg-density, and EPI

Fungi previously isolated from the SCN using culture-dependent methods predominantly belong to Ascomycota. These include nematode-trapping fungi from the order Orbiliales such as the genera *Arthrobotrys, Dactylella*, and *Monacrosporium* (Swe et al., 2011). Many egg-parasites and J2 endoparasites belong to the Sordariomycete order Hypocreales, which contains a large number of both nematode and insect parasites (Sung et al., 2007). The most frequently isolated taxa from the J2 of SCN are obligate endoparasites such as *H. rhossiliensis* and *H. minnesotensis* (Liu and Chen, 2000). Fungi frequently isolated from females and cysts of the SCN have included many taxa from Ascomycota, including members of Hypocreales [*Neocosmospora vasinfecta, Fusarium solani, F. oxysporum, P. chlamydosporia* (Chen et al., 1996; Liu and Chen, 2003), *Purpureocillium lilacinum* (Chen et al., 1996), *C. rosea, Cylindrocarpon destructans*, and *C. olidum]* as well as taxa from other ascomycete groups *(Dictyochaeta coffeae, D. heteroderae, Pyrenochaeta terrestris, Exophiala pisciphila, Stagonospora heteroderae*, and *Phoma heteroderae* among others) (Morgan-Jones et al., 1981; Gintis and Morgan Jones, 1982; Carris et al., 1989; Chen et al., 1994; Chen and Chen, 2002). Several have also been extensively studied for their biological control potential, including *P. chlamydosporia* (Chen et al., 1996; Liu and Chen, 2003), *P. lilacinum* (Chen et al., 1996), *E. pisciphila* (Chen and Chen, 2002), *Lecanicillium lecanii*, and a filamentous non-sporulating fungus tentatively named ARF18 (Kim and Riggs, 1991).

The results of our metabarcoding approach showed that relative abundance of fungal taxa in the cysts changed over time during the growing season. Among Ascomycota, Sordariomycetes, which included many of these potential egg and J2 parasites within Hypocreales, were increased at midseason. Among these, the known biocontrol fungus *P. chlamydosporia* had the highest relative abundance, while less abundant taxa included J2 pathogens such as *Hirsutella* spp. and *Haptocillium* spp. In contrast, the Dothideomycetes, a diverse class of ascomycetes that includes a number of key plant pathogens (*Cochliobolus* spp., *Alternaria* spp., and *Leptosphaeria* spp.), as well as endophytic and plant saprobic taxa (Schoch et al., 2009; Ohm et al., 2012), were decreased at midseason. Similarly, the Orbiliomycetes, which contain most of the genera of nematode trapping fungi, were also decreased at midseason. Most nematode trapping fungi can also grow as saprotrophs in soil (Liu et al., 2009) and only form trapping devices when they detect the mobile J2 stage in soil (Liu et al., 2009). They have been shown to be enriched in the rhizosphere and to colonize plant roots (Bordallo et al., 2002). A possible explanation for a decrease in nematode trapping fungi in cysts at midseason is that carbon resources for saprobic growth, as well as increased availability of roots and juvenile nematodes for colonization, all promote growth of trapping fungi in soil.

Several classes of ascomycetes (Eurotiomycetes, Sordariomycetes, and Orbiliomycetes) also showed higher relative abundance in soybean than corn crop sequences and in first year of corn following either a 5 year or annual rotation of soybean (Figure 6). Previous research has shown that some nematophagous fungi, particularly obligate taxa, show a density dependent population dynamic, increasing in abundance with higher densities of their host (Jaffee et al., 1992; Jaffee, 1993). Although we did not find a significant correlation with SCN egg-density and year of soybean, a previous study at this site found that the total nematode population in soybean plots was higher than in the corn plots (Grabau and Chen, 2016b) and our results do show a trend of increasing egg-density with increasing years of soybean (Figure 2). The increased relative abundance of nematophagous fungi in soybean crop sequences may be due to higher nematode populations supporting greater numbers of nematophagous fungi (Jaffee, 1993). The Sordariomycetes in particular showed significantly higher relative abundance in longer soybean crop sequences and continuous monoculture (Figure 6). Close inspection at the genus level showed that the known Sordariomycete biocontrol fungus *Pochonia*, as well as genera in Dothideomycetes (*Leptosphaeria)* and Eurotiomycetes (*Exophiala)*,were consistently and positively correlated with increasing years of continuous soybean monoculture. The results of an earlier study (Hu et al., 2017) showed that *Pochonia* was decreased in cysts in a field with biocidal treatment, suggesting the important role of this genus in regulating SCN populations. *Leptosphaeria* has not previously been widely reported from SCN cysts using culture-dependent methods (Chen and Chen, 2002) and its potential role in cysts remains unknown but warrants further research. *Exophiala* has been commonly isolated from cysts and shown to be able to parasitize eggs (Table S1). Several other genera in Hypocreales previously isolated from cysts, including *Geosmithia, Cylindrocarpon*, and *Nectria*, were also significantly positively correlated with EPI, supporting their role in parasitizing eggs (Tables S1, S10).

An unexpected finding in our study was that the percentage of OTUs of several early diverging groups of fungi, typically considered to be plant symbionts, plant-associates, or saprotrophs, also changed over the growing season. The percentage of OTUs mapping to Glomeromycotina (e.g., AMF) was consistently increased in the fall. As more obligate symbionts of roots and of limited saprobic capability, the Glomeromycotina may take longer to colonize roots systems and invade cysts. Another possibility is that some species of AMF are actually parasitic to SCN. One species, *Glomus fasciculatum*, in fact, has previously been shown to sporulate in SCN cysts and infect eggs (Francl and Dropkin, 1985). However, studies of the biocontrol efficacy of AMF and the interactions of AMF and SCN have shown equivocal results. Previous studies have shown suppression of SCN reproduction and colonization early in the season, potentially due to competition for space and resources within the root, but soybean roots attained nearly equal densities of SCN when grown to maturity (Tylka et al., 1991; Price et al., 1995; Todd et al., 2001). However, most of these studies were limited to only a few species of AMF. Given differences in symbiotic interactions with plants among different species of AMF, the composition of species comprising the AMF community is likely to have a strong effect on the potential function of AMF in biocontrol of the SCN.

The proportion of OTUs belonging to the phyla Mortierellomycotina and the class Mortierellomycotina_cls_Incertae_sedis both increased at midseason. Fungi belonging to Mortierellomycotina are very common in soil (Benny et al., 2014) and many are also known to be plant-associated either as rhizosphere-associates, root endophytes, or litter inhabitants (Cha et al., 2011; Murase et al., 2012; Peltoniemi et al., 2015). Some species within genus *Mortierella* also harbor symbiotic bacteria that may contribute to functions of these fungi in ecosystems (Wani et al., 2017). Although several taxa from the Mortierellomycotina, including an unidentified *Mortierella* spp. (Chen and Chen, 2002) and a new species and genus closely related to *Mortierella, Echinochlamydosporium variabile* (Jiang et al., 2011), have been previously isolated from the SCN, reports of this group parasitizing nematodes are rare in the literature. Our pipeline identified 7 OTUs assigned to putative species of *Mortierella* including *M. alpina, Mortierella ambigua, Mortierella amoeboidea, M. elongata, Mortierella exigua, Mortierella hyalina*, and one unidentified *Mortierella* spp. (Tables S4, 10). The genus *Mortierella* was also significantly correlated with increasing years of soybean monoculture (Figure 7), and two species (*M. alpina* and *M. elongata*) were also correlated with egg-density in spring of 2016 (Table S10). While we suggest caution in confidently assigning OTUs to the species level, the presence of 7 distinct OTUs belonging to *Mortierella* suggests that further investigation of this group as potential nematode parasites is warranted. One of the putative species detected among our OTUs and shown to be positively correlated with egg-density, *M. alpina*, has previously been tested and shown to have biocontrol potential, including reduced egg hatch, juvenile mortality, and disease severity in other nematodes such as the root knot nematode (Al-Shammari et al., 2013). In this study, *M. elongata* was also found to be strongly correlated with egg-density, supporting its role as a potential egg-parasite. Interestingly, some Mortierellomycotina may be able to outcompete or exclude other egg-parasitic fungi in pre-colonized cysts as one species of *Mortierella* colonizing cysts was shown to reduce the ability of *P. chlamydosporia* to invade cysts (Chen and Chen, 2003).

One additional species of early diverging fungal taxa, *H. montanus* in Kickxellomycotina, was also found to be strongly positively correlated with egg-density in spring and midseason of 2015 (Table S10). Other Harpellales have previously been isolated as symbionts of dipteran insects (White et al., 2006). This taxa was also a predominant member of the Animal Endosymbiont FUNGuild, which was also strongly correlated with EPI in spring and fall of 2015. This data suggests a possible association of *Harpellomyces* with nematodes as well as insects.

### Changes in fungal ecological guilds

It is not surprising that the majority of fungi could not be confidently assigned to known functional guilds, as there is limited data on fungal ecologies and nutritional strategies, and such data is needed to make assignments in the FUNGuild database (Nguyen et al., 2016). There are also fungi that may function in multiple roles in an ecosystem, resulting in multiple guild assignments to a single fungus. The majority of OTUs were assigned to either guilds Undefined Saprotroph or Plant Pathogen. Saprotrophs generally grow faster and can utilize more nutritional resources from soil or plant debris when competing with parasites, especially obligate parasitic fungi of nematodes, whose growth and development are often limited by availability of a suitable host (Chen and Chen, 2003). Many saprotrophic fungi have also been observed colonizing SCN cysts and could protect the eggs from being colonized by parasitic fungi to some degree (Chen and Chen, 2003). The Undefined Saprotroph category contained all the OTUs assigned to Mortierellomycotina as well as a diversity of other taxa (Table S11). Our data showed an increase in the Undefined Saprotroph category as well as the taxonomic group, Mortierellomycotina, at midseason. The presence of *Mortierella* spp. within cysts and previous reports of several members parasitizing nematode eggs raises the question of whether some member of this genus could also function as Animal Pathogens. Guilds containing the Animal Pathogen functional group are relevant to nematode parasitism (Bird, 1980). The Animal Pathogen-Endophyte-Fungal Parasite-Lichen Parasite-Plant Pathogen-Wood Saprotroph guild was significantly increased in relative abundance at midseason (Figure 8A; Table S12). This guild contained many fungi in the family Nectriacaea that have previously been isolated from SCN cysts (Tables S1, S11). In this study, the genus *Nectria* was also found to be correlated with high EPI (Table S13). The Animal Pathogen guild, which included several known egg-parasites such as *P. chlamydosporia* as well as other hypocrealean endoparasitic genera including *Haptocillium* spp. and *Hirsutella* spp. (Table S11), also increased during midseason, although not significantly (Figure 8A; Table S12).

### Nematophagous fungal guilds

In addition to the FUNGuild comparison, we also defined nematophagous fungal guilds based on the literature (Table S1). Although it can be difficult to assign specific taxa to a particular group of nematode parasites due to a paucity of studies testing the pathogenicity of fungal isolates to specific nematode life stages and the fact that some fungi may have multiple trophic modes, we used literature searches to assign fungi with substantial evidence of parasitism or a specific life stage to nematophagous guilds. Our results validated that trapping fungi and endoparasitic fungi have low relative abundance in SCN cysts, which is likely because cysts contain mostly eggs rather than second stage juveniles, the life stage consumed by these two guilds. In the cysts, there may be a small proportion of hatched second stage juveniles which harbor small amounts of trapping and endoparasitic fungi. One of the challenges of identifying egg-parasites is that many saprotrophic fungi have also been isolated from SCN cysts. Whether or not they can actively parasitize live eggs or only feed on dead eggs within cysts is not always known, although a number have been tested in bioassays and demonstrated to be capable of both penetrating cyst walls and parasitizing live eggs inside of cysts (Chen and Dickson, 1996; Chen et al., 1996). The egg-parasitic fungi in our study were classified based on strict criteria including (1) previous reports of isolation directly from SCN eggs, and (2) demonstrated ability to parasitize SCN eggs in bioassays or a high EPI (Meyer et al., 1990, 2004; Chen et al., 1996, 2000; Chen and Chen, 2002, 2003) (Table S1). Thus, these fungi are more likely to be true egg-parasites. Our results showed that this egg-parasitic fungal guild showed a trend of increasing relative abundance with increasing years of continuous soybean.

## Conclusions

Our study is one of the first to explore the fungal community inhabiting the unique microenvironment of the SCN cyst as affected by long-term crop rotation. Looking across three seasons over 2 years, our data showed clear changes in relative abundance of several groups of fungi including those with known egg-parasitic activity and several novel groups that may also have roles in nematode parasitism. Overall species diversity increased in the first year of crop rotation after either an annual or 5 year rotation as well as over the growing season and was the greatest in fall. Known egg-parasitic taxa such as *P. chlamydosporia* as well as ecological guilds containing an animal pathogen lifestyle and several taxa within Mortierellomycotina consistently increased in relative abundance at midseason. The relative abundance of known and candidate biocontrol genera (e.g., *Pochonia* and *Exophiala*), as well as several new candidate egg-parasitic genera (e.g., *Leptosphaeria* and *Mortierella*), showed a positive correlation with increasing years of soybean monoculture. Our study also suggests that several early diverging groups of fungi that were strongly correlated with either egg-density (*M. alpina, M. elongata*, and *H. montanus*) may also have roles as either nematode egg-parasites or symbionts. Crop treatment effects and measures of EPI appeared stronger at midseason when egg-parasitic taxa have the highest relative abundance. In contrast, the AMF, which have also been suggested to have a potential interaction with the SCN, increased in relative abundance in the fall. This study contributes to our understanding of the effects of seasonal variation and crop rotation on fungal diversity in the SCN cysts and provides fundamental knowledge of the ecology and dynamics of nematode parasitic fungi in agroecosystems in order to advance the use of egg-parasitic fungi for the biological control of destructive nematode pests.

## Author contributions

KB and SC: conceived of and supervised the research; WH: performed the research, isolating DNA, and metabarcode sequencing of samples, designed metabarcoding analysis pipelines, and analyzed and interpreted the data; NS: contributed data analysis pipelines and performed literature searches for classification of nematophagous fungi; DH: assisted in collecting cyst samples, synthesized literature on egg-parasitic fungi, provided images for figures, and edited the manuscript; WH: wrote the manuscript with KB and editing assistance from SC, NS, and DH.

### Conflict of interest statement

The authors declare that the research was conducted in the absence of any commercial or financial relationships that could be construed as a potential conflict of interest.

## Supplementary material

Figure S1 Rarefaction curve across all samples.

Figure S2 The relative abundance of FUNGuilds affected by crop sequences.

Table S1 Fungal taxa assigned to nematophagous fungal guilds.

Table S2 Statistical results of ANOVA of egg population density across crop sequences.

Table S3 Correlation between SCN egg density and EPI.

Table S4 OTU table with taxonomic assignments from UNITE.

Table S5 Fungal phyla and classes that differ significantly across season.

Table S6 Procrustes test of Bray–Curtis dissimilarity between seasons.

Table S7 Beta dispersion parameters of NMDS analysis of fungal communities across crop sequences.

Table S8 Fungal genera that differ significantly across crop sequences.

Table S9 Correlation of fungal genera with increasing years of soybean crop sequences.

Table S10 Fungal taxa correlated with SCN egg density and EPI.

Table S11 Fungal taxa found in FUNGuild categories.

Table S12 Statistical results of ANOVA of fungal guilds across seasons.

Table S13 FUNGuild categories correlated with SCN egg density and EPI.
