# Supplementary figures and images for: Mycobiome of Cysts of the Soybean Cyst Nematode Under Long Term Crop Rotation
Source: Front Microbiol. 2018 Mar 16;9:386. doi: 10.3389/fmicb.2018.00386 (PMC5865410; doi:10.3389/fmicb.2018.00386)

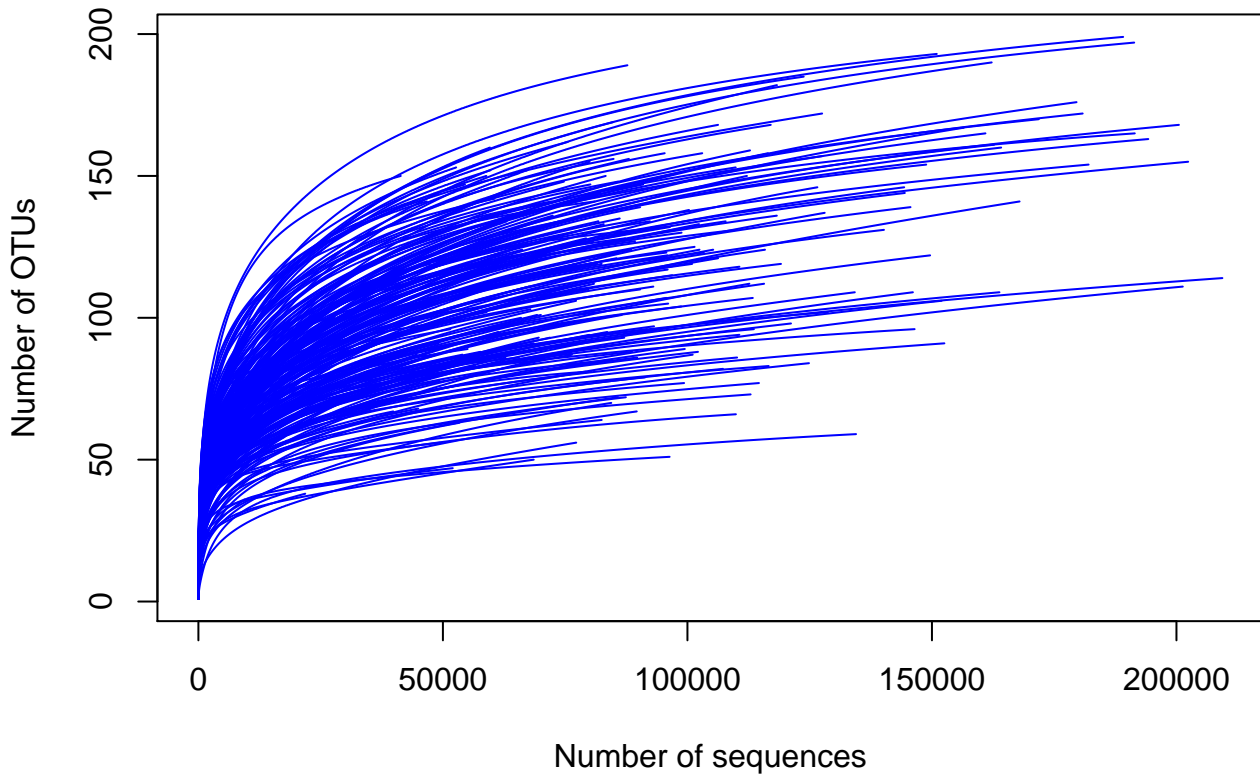

Supplement: Figure S1 — Rarefaction curve across all samples. [file Image1.PDF]

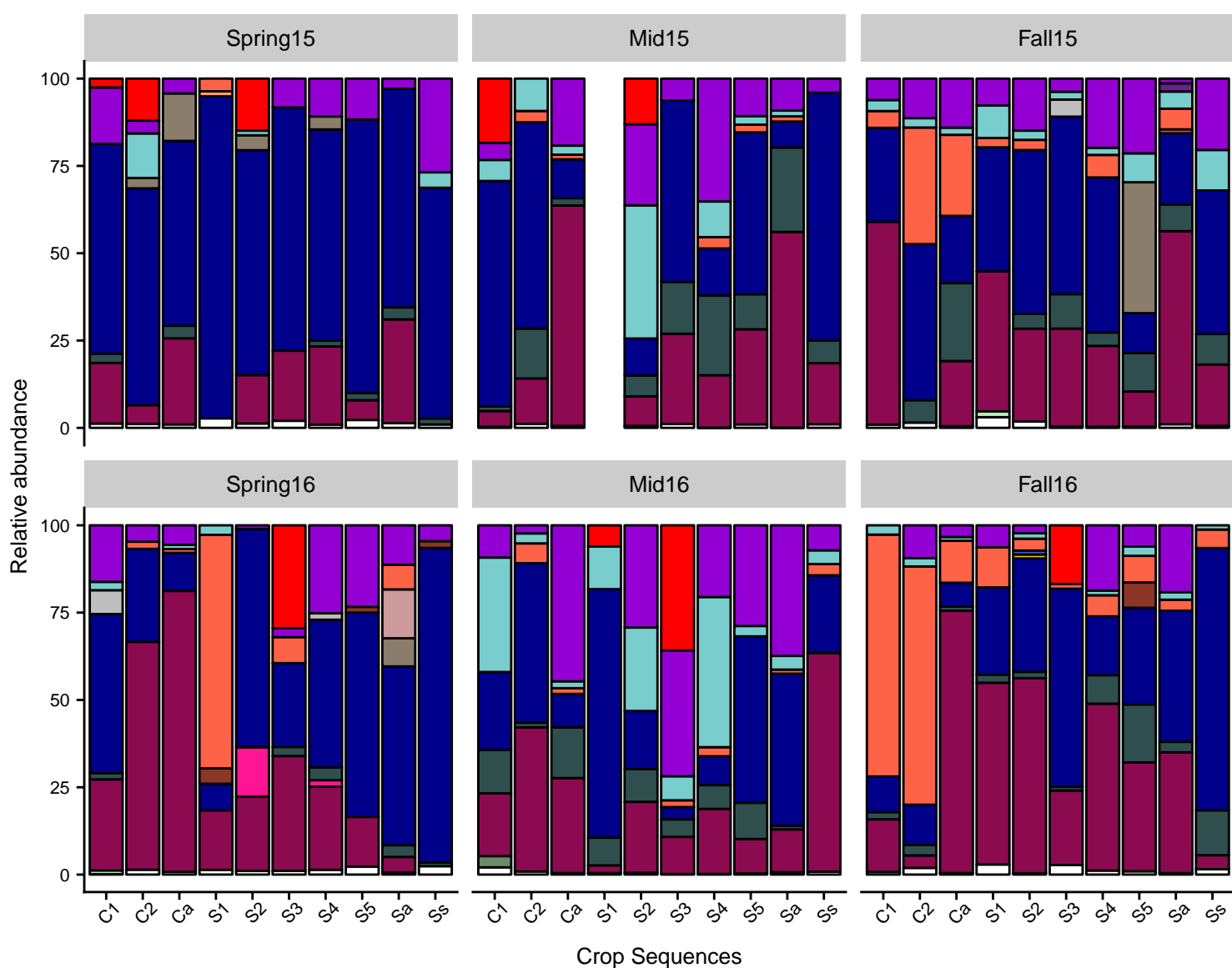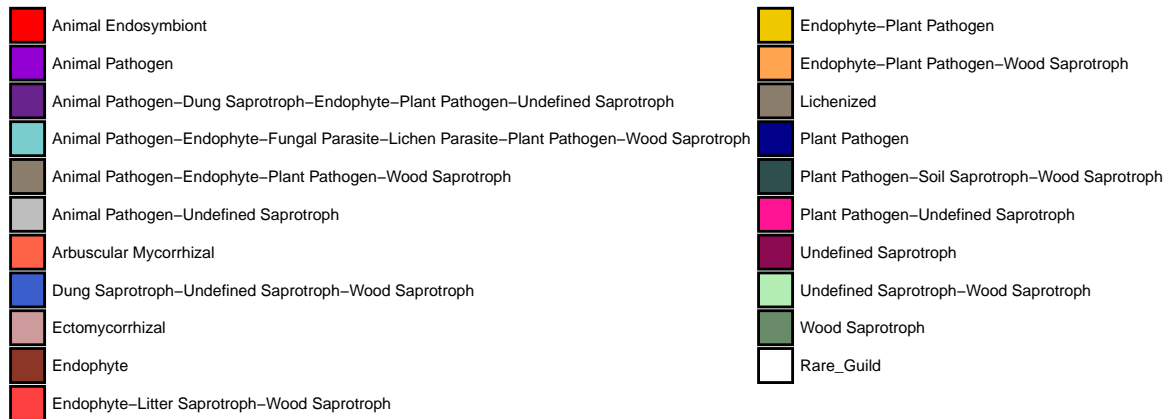

Supplement: Figure S2 — The relative abundance of FUNGuilds affected by crop sequences. [file Image2.PDF]
