# Supplementary material for: Mycobiome of Cysts of the Soybean Cyst Nematode Under Long Term Crop Rotation
Source: Front Microbiol. 2018 Mar 16;9:386. doi: 10.3389/fmicb.2018.00386 (PMC5865410; doi:10.3389/fmicb.2018.00386)
Supplement: Table S1 — Fungal taxa assigned to nematophagous fungal guilds. [file Table1.DOCX]

**STable 1**. Fungi isolated from SCN (1-5) and assignment to nematophagous fungal guild.

| Genus | Species | Synonymns | TrueEggParasites | Nematophagous Fungal Guild |
| --- | --- | --- | --- | --- |
| *Arthrobotrys* |  |  |  | nematode_trapping |
| *Dactylaria* |  |  |  | nematode_trapping |
| *Dactylella* |  |  |  | nematode_trapping |
| *Monacrosporium* |  |  |  | nematode_trapping |
| *Drechslerella* |  |  |  | nematode_trapping |
| *Dactylellina* |  |  |  | nematode_trapping |
| *Dactylella* | *oviparasitica* | |  | nematode_trapping |
| *Arthrobotrys* | *dactyloides* | |  | Nematode trapping |
| *Hirsutella* | *minnesotensis* | |  | endoparasite |
| *Hirsutella* | *rhossiliensis* | |  | endoparasite |
| *Acremonium* | sp |  |  | egg_parasite |
| *Cylindrocarpon* | sp |  | Yes | egg_parasite |
| *Epicoccum* | *nigrum* |  |  | egg_parasite |
| *Mortierella* | sp |  |  | egg_parasite |
| *paraphoma* | *radicina* |  |  | egg_parasite |
| *Fusarium* | *equiseti* |  |  | egg_parasite |
| *Fusarium* | *oxysporum* | |  | egg_parasite |
| *Cylindrocarpon* | *destructans* | | Yes | egg_parasite |
| *Clonostachys* | sp |  |  | egg_parasite |
| *Pyrenochaeta* | *terrestris* |  | Yes | egg_parasite |
| *Metacordyceps* | *chlamydosporia* | | Yes | egg_parasite |
| *Purpureocillium* | *lilacinus* |  | Yes | egg_parasite |
| *Fusarium* | *solani* |  |  | egg_parasite |
| *Phoma* | sp |  | Yes | egg_parasite |
| *Nematophthora* | *gynophila* | |  | egg_parasite |
| *Verticillium* | *lecanii* |  | Yes | egg_parasite |
| *Leptosphaeria* | sp |  |  | egg_parasite |
|  |  | |  |  |
| *Stagonospora* | *heteroderae* | |  | egg_parasite |
| *Neocosmospora* | *vasinfecta* | |  | egg_parasite |
| *Exophiala* | *pisciphila* |  |  | egg_parasite |
| *Phoma* | *chrysanthemicola* | |  | egg_parasite |
| *Trichoderma* | *polysporum* | |  | egg_parasite |
| *Acremonium* | *bacillisporum* | |  | egg_parasite |
| *Drechmeria* | *coniospora* | |  | egg_parasite |
| *Scytalidium* | *fulvum* |  |  | egg_parasite |
| *Thielaviopsis* | *basicola* |  |  | egg_parasite |
| *Corynespora* | *cassiicola* |  |  | egg_parasite |
| *Oidiodendron* | *cerealis* |  |  | egg_parasite |
| *Rhizoctonia* | sp |  |  | egg_parasite |
| *Pythium* | sp |  |  | egg_parasite |
| *Gliocladium* | sp |  |  | egg_parasite |
| *Staphylotrichum* | sp |  |  | egg_parasite |
| *Clonostachys* | *rosea* | Gliocladium roseum | Yes | egg_parasite |
| *Idriella* | sp |  |  | egg_parasite |

**Literature Cited**

1. S. L. F. Meyer, R. N. Huettel, X. Z. Liu, R. A. Humber, J. Juba and J. K. Nitao: Activity of fungal culture filtrates against soybean cyst nematode and root-knot nematode egg hatch and juvenile motility. *Nematology*, 6(1), 23-32 (2004)

2. S. Y. Chen and F. J. Chen: Fungal parasitism of *Heterodera glycines* eggs as influenced by egg age and pre-colonization of cysts by other fungi. *Journal of Nematology*, 35(3), 271-277 (2003)

3. G. R. Stirling: Biological Control of Plant-parasitic Nematodes: Soil Ecosystem Management in Sustainable Agriculture, 2nd Edition. (2014) doi:10.1079/9781780644158.0000

4. S. Y. Chen, D. W. Dickson and D. J. Mitchell: Pathogenicity of fungi to eggs of *Heterodera glycines*. *Journal of Nematology*, 28(2), 148-158 (1996)

5. B. R. Kerry, D. H. Crump and L. A. Mullen: Studies of the cereal cyst nematode, Heterodera avenae under continuous cereals, 1975–1978. II. Fungal parasitism of nematode females and eggs. *Annals of Applied Biology*, 100(3), 489-499 (1982)
