# Supplementary material for: Mycobiome of Cysts of the Soybean Cyst Nematode Under Long Term Crop Rotation
Source: Front Microbiol. 2018 Mar 16;9:386. doi: 10.3389/fmicb.2018.00386 (PMC5865410; doi:10.3389/fmicb.2018.00386)
Supplement: Table S2 — Statistical results of ANOVA of egg population density across crop sequences. [file Table2.DOCX]

**STable 2**. ANOVA result of the egg population density.

| ANOVA | 2015 |  | 2016 |
| --- | --- | --- | --- |
| CropSeq | 0.026* |  | 0.29 |
| Season | 0.99 |  | 0.03* |
| CropSeq*Season | 0.84 |  | 0.65 |
