# Supplementary material for: Mycobiome of Cysts of the Soybean Cyst Nematode Under Long Term Crop Rotation
Source: Front Microbiol. 2018 Mar 16;9:386. doi: 10.3389/fmicb.2018.00386 (PMC5865410; doi:10.3389/fmicb.2018.00386)
Supplement: Table S3 — Correlation between SCN egg density and EPI. [file Table3.DOCX]

STable 3. Correlation of EPI with crop sequence treatment and egg density

| Season | Crop Sequence  P-value | Egg Density  P value | Egg Density  Equation |
| --- | --- | --- | --- |
| Mid15 | 0.46 | 0.46 |  |
| Fall15 | 0.16 | 0.004** | Y = 433.4 + 1589X |
| Mid16 | 0.74 | 0.29 |  |
| Fall16 | 0.57 | 0.56 |  |
