# Supplementary material for: Mycobiome of Cysts of the Soybean Cyst Nematode Under Long Term Crop Rotation
Source: Front Microbiol. 2018 Mar 16;9:386. doi: 10.3389/fmicb.2018.00386 (PMC5865410; doi:10.3389/fmicb.2018.00386)
Supplement: Table S5 — Fungal phyla and classes that differ significantly across season. [file Table5.DOCX]

**STable 5.**  A) Phyla and B) Classes significantly different across season at FDR adjusted *P* values < 0.05 (*), < 0.01 (**),and <0.001 (***). Letters indicate Tukey’s test with the same letter indicating no significant difference at *P* < 0.05.

|  | 2015 |  |  |  | 2016 |  |  |  |
| --- | --- | --- | --- | --- | --- | --- | --- | --- |
| 1. Phylum | Spring | Mid | Fall | *P* value | Spring | Mid | Fall | *P* value |
| Ascomycota | a | b | a | < 0.001*** | a | c | b | 0.002** |
| Mortierellomycotina | b | a | a | < 0.001*** | b | a | a | 0.02* |
| Chytridiomycota | a | b | b | 0.002** | a | a | a | 0.44 |
| Glomeromycotina | b | b | a | < 0.001*** | b | b | a | < 0.001*** |
| Basidiomycota | a | ab | b | 0.04* | a | a | a | 0.98 |
| Unidentified | b | a | b | 0.008** | c | a | b | < 0.001*** |
| 1. Class |  |  |  |  |  |  |  |  |
| Sordariomycetes | b | a | a | 0.03* | c | a | b | < 0.001*** |
| Mortierellomycotina_cls_Incertae_sedis | b | a | a | < 0.001*** | b | a | a | 0.03* |
| Glomeromycetes | b | b | a | 0.01* | b | b | a | < 0.001*** |
| Orbiliomycetes | a | a | a | 0.18 | a | b | a | < 0.001*** |
| Dothideomycetes | a | b | c | < 0.001*** | a | b | ab | 0.004** |
| Pezizomycotina_cls_Incertae_sedis | a | a | a | 0.51 | a | a | a | 0.079 |
| Leotiomycetes | a | a | a | 0.18 | a | b | a | 0.004** |
| Eurotiomycetes | a | a | a | 0.12 | a | b | a | <0.001*** |
| 1. Genera |  |  |  |  |  |  |  |  |
| Mortierella | b | a | b | 0.012* | a | a | a | 0.7 |
| Leptosphaeria | a | b | b | <0.001*** | a | a | a | 0.6 |
| Nectria | a | a | a | 0.6 | b | a | b | 0.002** |
| Exophiala | a | a | a | 0.6 | b | b | a | 0.001*** |
|  |  |  |  |  |  |  |  |  |
|  |  |  |  |  |  |  |  |  |
