# Supplementary material for: Mycobiome of Cysts of the Soybean Cyst Nematode Under Long Term Crop Rotation
Source: Front Microbiol. 2018 Mar 16;9:386. doi: 10.3389/fmicb.2018.00386 (PMC5865410; doi:10.3389/fmicb.2018.00386)
Supplement: Table S6 — Procrustes test of Bray–Curtis dissimilarity between seasons. [file Table6.DOCX]

**STable 6**. Procrustes test of Bray-Curtis dissimilarity between seasons and FDR adjusted *P* values 0.05 (*), < 0.01 (**), and <0.001 (***).

|  | 2015 | |  |  | 2016 | |  |
| --- | --- | --- | --- | --- | --- | --- | --- |
| Community | Spring vs. Mid | Mid vs. Fall | Fall vs. Spring |  | Spring vs. Mid | Mid vs. Fall | Fall vs. Spring |
| Overall | 0.002** | 0.001 ** | 0.016* |  | 0.001** | 0.001** | 0.001** |
| C1 | 0.21 | 0.14 | 0.1 |  | 0.57 | 0.83 | 0.19 |
| C2 | 0.095 | 0.33 | 0.14 |  | 0.095 | 0.25 | 0.14 |
| Ca | 0.33 | 0.08 | 0.29 |  | 0.76 | 0.17 | 0.81 |
| S1 | NA | NA | NA |  | 0.86 | 0.58 | 0.19 |
| S2 | 0.23 | 0.81 | 0.43 |  | 0.81 | 0.17 | 0.48 |
| S3 | 0.29 | 0.54 | 0.67 |  | 0.095 | 0.13 | 0.05 |
| S4 | 0.048* | 0.13 | 0.43 |  | 0.19 | 0.63 | 0.05 |
| S5 | 0.23 | 0.75 | 0.9 |  | 0.81 | 0.25 | 0.76 |
| Sa | 0.62 | 0.58 | 0.14 |  | 0.095 | 0.54 | 0.9 |
| Ss | 0.86 | 0.71 | 0.58 |  | 0.048* | 0.71 | 0.9 |
