# Supplementary material for: Mycobiome of Cysts of the Soybean Cyst Nematode Under Long Term Crop Rotation
Source: Front Microbiol. 2018 Mar 16;9:386. doi: 10.3389/fmicb.2018.00386 (PMC5865410; doi:10.3389/fmicb.2018.00386)
Supplement: Table S7 — Beta dispersion parameters of NMDS analysis of fungal communities across crop sequences. [file Table7.DOCX]

| **STable 7.**  Beta dispersion parameters of NMDS analysis of fungal community across crop sequences. | | | | | | | | | | | | | | | | | | |
| --- | --- | --- | --- | --- | --- | --- | --- | --- | --- | --- | --- | --- | --- | --- | --- | --- | --- | --- |
|  | 2015 | | | | | |  |  | 2016 | | | | | | | | | |
|  | Spring |  | Mid | | | Fall |  | Spring | | |  | Mid | | | Fall | | | |
| Crop Seq |  |  | | | |  |  |  | |  | | | | | | |  | |
| C1 | 0.42 | abc | | 0.46 | ab | 0.40 | abc |  | 0.35 | | d | | 0.46 | ab | | 0.45 | | ab |
| C2 | 0.39 | bc | | 0.40 | ab | 0.46 | a |  | 0.46 | | abc | | 0.51 | a | | 0.51 | | a |
| Ca | 0.47 | ab | | 0.48 | ab | 0.44 | ab |  | 0.36 | | cd | | 0.39 | ab | | 0.39 | | abc |
| S1 | 0.53 | a | | NA |  | 0.35 | abc |  | 0.52 | | a | | 0.46 | ab | | 0.39 | | abc |
| S2 | 0.43 | abc | | 0.52 | a | 0.41 | abc |  | 0.47 | | abc | | 0.36 | b | | 0.32 | | c |
| S3 | 0.44 | abc | | 0.36 | b | 0.38 | abc |  | 0.44 | | abcd | | 0.42 | ab | | 0.41 | | abc |
| S4 | 0.37 | bc | | 0.38 | b | 0.29 | c |  | 0.36 | | cd | | 0.44 | ab | | 0.36 | | bc |
| S5 | 0.34 | c | | 0.40 | ab | 0.35 | abc |  | 0.34 | | d | | 0.37 | ab | | 0.31 | | c |
| Sa | 0.36 | bc | | 0.41 | ab | 0.39 | abc |  | 0.50 | | ab | | 0.44 | ab | | 0.37 | | bc |
| Ss | 0.42 | abc | | 0.40 | ab | 0.39 | abc |  | 0.41 | | bcd | | 0.46 | ab | | 0.39 | | bc |
| *P* value | 0.002** | abc | | 0.02* | ab | 0.004** | abc |  | <0.001*** | | d | | 0.037* | ab | | <0.001*** | | ab |
| FDR adjusted *P-*values for ANOVA comparing beta-dispersion parameters at 0.05 (*), < 0.01 (**), and <0.001 (***). The beta-dispersion value is the mean of distance to centroid across season and crop sequences. | | | | | | | | | | | | | | | | | | |
