# Supplementary material for: Mycobiome of Cysts of the Soybean Cyst Nematode Under Long Term Crop Rotation
Source: Front Microbiol. 2018 Mar 16;9:386. doi: 10.3389/fmicb.2018.00386 (PMC5865410; doi:10.3389/fmicb.2018.00386)
Supplement: Table S8 — Fungal genera that differ significantly across crop sequences. [file Table8.DOCX]

**STable 8**. Genera significantly different across all crop sequences by ANOVA.

| Fall15 | | |  | Fall16 | | | |  |
| --- | --- | --- | --- | --- | --- | --- | --- | --- |
| *Phadidium* | |  |  | *Exophiala* | |  | *Malassezia* | |
| C1 | 0.14 | b |  | 0.07 | d |  | 0.00 | bc |
| C2 | 2.46 | a |  | 0.15 | d |  | 0.03 | a |
| Ca | 0.09 | b |  | 1.17 | cd |  | 0.00 | bc |
| S1 | 0.13 | b |  | 3.37 | a |  | 0.00 | bc |
| S2 | 0.04 | b |  | 1.95 | bc |  | 0.00 | bc |
| S3 | 0.00 | b |  | 1.33 | cd |  | 0.00 | bc |
| S4 | 0.00 | b |  | 1.06 | cd |  | 0.00 | bc |
| S5 | 0.00 | b |  | 1.88 | bc |  | 0.01 | bc |
| Sa | 0.00 | b |  | 2.84 | ab |  | 0.00 | c |
| Ss | 0.01 | b |  | 0.11 | d |  | 0.01 | b |

The value is the mean relative abundance (across n=4 replicate plots) of genera

and columns with different letters indicate significant differences (*P-*value= 0.05)

across crop sequences according Tukey’s and LSD test.
