# Supplementary material for: Mycobiome of Cysts of the Soybean Cyst Nematode Under Long Term Crop Rotation
Source: Front Microbiol. 2018 Mar 16;9:386. doi: 10.3389/fmicb.2018.00386 (PMC5865410; doi:10.3389/fmicb.2018.00386)
Supplement: Table S9 — Correlation of fungal genera with increasing years of soybean crop sequences. [file Table9.DOCX]

**STable 9**. Genera significantly correlated with soybean crop sequence year. *P* value, R square value, and best fit linear regression line are shown.

| Genera | Season | Equation | R square | *P* value |
| --- | --- | --- | --- | --- |
| *Leptosphaeria* | Spring15 | Y = 1.44 + 0.52x | 0.18 | 0.04* |
| *Pochonia* | Spring15 | Y = -0.51 + 0.49x | 0.31 | 0.009** |
| *Mortierella* | Spring15 | Y = 0.15 + 0.12x | 0.2 | 0.04* |
| *Exophiala* | Mid15 | Y = -0.21 + 0.4x | 0.15 | 0.08. |
| *Leptosphaeria* | Mid15 | Y = -0.96 + 0.67x | 0.29 | 0.02* |
| *Arthrobotrys* | Fall15 | Y = -0.002 + 0.001x | 0.17 | 0.04* |
| *Conlarium* | Fall15 | Y = -0.0004 + 0.0002x | 0.15 | 0.05* |
| *Lachnum* | Fall15 | Y = 2.1 + -0.4x | 0.17 | 0.04* |
| *Leptosphaeria* | Spring16 | Y = 0.54 + -0.14x | 0.18 | 0.04* |
| *Pochonia* | Spring16 | Y = -0.03 + 0.002x | 0.15 | 0.05* |
| *Mortierella* | Spring16 | Y = -0.53 + 0.75x | 0.42 | 0.001*** |
| *Acaulospora* | Mid16 | Y = -0.02 + 0.014x | 0.17 | 0.04* |
| *Leptosphaeria* | Mid16 | Y = -0.43 + 0.36x | 0.38 | 0.002** |
| *Exophiala* | Fall16 | Y = 3.1 + -0.39x | 0.18 | 0.04* |
| *Trichometasphaeria* | Fall16 | Y = 0.007 + -0.002x | 0.18 | 0.04* |
