# Supplementary material for: Mycobiome of Cysts of the Soybean Cyst Nematode Under Long Term Crop Rotation
Source: Front Microbiol. 2018 Mar 16;9:386. doi: 10.3389/fmicb.2018.00386 (PMC5865410; doi:10.3389/fmicb.2018.00386)
Supplement: Table S10 — Fungal taxa correlated with SCN egg density and EPI. [file Table10.DOCX]

**STable 10**. Fungal Taxa significantly correlated with SCN egg density and EPI.

| 1. Fungal taxa significantly correlated with SCN egg density | | | | |
| --- | --- | --- | --- | --- |
| Taxa | Season | Equation | R square | Adjusted *P* value |
| Harpellomyces_montanus | Spring15 | Y = -0.39 + 0.0005X | 0.31 | < 0.001*** |
| Neophaeosphaeria_agaves | Spring15 | Y= -0.27 + 0.0005X | 0.56 | 0.02* |
| Sarcosomataceae_unidentified | Mid15 | Y = -0.0004 + 0.000005X | 0.26 | 0.04* |
| Phanerochaete_unidentified | Mid15 | Y = -0.004 + 0.0004X | 0.26 | 0.04* |
| Neophaeosphaeria_agaves | Mid15 | Y = -0.44 + 0.0005X | 0.36 | <0.001*** |
| Harpellomyces_montanus | Mid15 | Y = -0.4 + 0.0005X | 0.39 | 0.006** |
| Parastagonospora_unidentified | Spring16 | Y = -0.002 + 0.00003X | 0.27 | 0.008** |
| Stagonospora_unidentified | Spring16 | Y = -0.1 + 0.0002X | 0.27 | 0.008** |
| Alternaria_unidentified | Spring16 | Y = -0.03 + 0.00009X | 0.29 | 0.008** |
| Neophaeosphaeria_agaves | Spring16 | Y = -0.28 + 0.0008X | 0.28 | 0.008** |
| Trichoderma_parapiluliferum | Spring16 | Y = -0.04 + 0.0001X | 0.27 | 0.008** |
| Phallus_unidentified | Spring16 | Y = -0.009 + 0.00005X | 0.27 | 0.008** |
| Microstromatales_unidentified | Spring16 | Y = -0.04 + 0.0001X | 0.27 | 0.008** |
| Rhizophlyctis_unidentified | Spring16 | Y = -0.09 + 0.0002X | 0.27 | 0.008** |
| Dentiscutata_erythropus | Spring16 | Y = -0.12 + 0.0003X | 0.34 | 0.005*** |
| Mortierella_alpina | Spring16 | Y = -0.02 + 0.00005X | 0.44 | <0.001*** |
| Mortierella_elongata | Spring16 | Y = -0.08 + 0.0003X | 0.24 | 0.02* |
| Eurotiales_unidentified | Mid16 | Y = -0.002 +0.000006X | 0.47 | <0.001*** |
| Diaporthe_phaseolorum | Mid16 | Y = -0.0006 + 0.000002X | 0.25 | 0.04* |
| Hannaella_oryzae | Mid16 | Y = -0.0008 + 0.000002X | 0.30 | 0.02* |
| 1. Fungal Taxa significantly correlated with SCN egg density and EPI | | | | |
| Taxa | Season | Equation | R square | Adjusted *P* value |
| Tubeufia unidentified | Mid16 | Y = -0.003+0.005X | 0.44 | 0.036* |
| Geosmithia unidentified | Mid16 | Y = -0.014+0.03X | 0.39 | 0.039* |
| Cylindrocarpon unidentified | Mid16 | Y = -0.03+0.06X | 0.41 | 0.036* |
| Nectria unidentified | Mid16 | Y = -0.5+1.9X | 0.46 | 0.036* |
| Bolbitiaceae unidentified | Mid16 | Y = -0.016+0.03X | 0.42 | 0.036* |
| Agaricales unidentified | Mid16 | Y = -0.04+0.09X | 0.41 | 0.036* |
| Blastocladiomycota unidentified | Mid16 | Y = -0.15+0.27X | 0.41 | 0.036* |
| Chytridiomycota unidentified | Mid16 | Y = -0.014+0.025X | 0.41 | 0.036* |
