# Supplementary material for: Mycobiome of Cysts of the Soybean Cyst Nematode Under Long Term Crop Rotation
Source: Front Microbiol. 2018 Mar 16;9:386. doi: 10.3389/fmicb.2018.00386 (PMC5865410; doi:10.3389/fmicb.2018.00386)
Supplement: Table S12 — Statistical results of ANOVA of fungal guilds across seasons. [file Table12.DOCX]

**STable 12.**  Guild significantly different across season at FDR adjusted *P* values < 0.05 (*), < 0.01 (**),and <0.001 (***). Letters indicate Tukey’s test with the same letter indicating no significant difference at *P* < 0.05.

|  | 2015 |  |  |  | 2016 |  |  |  |
| --- | --- | --- | --- | --- | --- | --- | --- | --- |
| Guild | Spring | Mid | Fall | *P* value | Spring | Mid | Fall | *P* value |
| Arbuscular Mycorrhizal | b | b | a | < 0.001*** | a | a | a | 0.45 |
| Plant Pathogen | a | b | b | 0.004** | a | a | a | 0.29 |
| Plant Pathogen-Soil Saprotroph-Wood Saprotroph | b | a | a | 0.03* | b | a | a | 0.04* |
| Animal Pathogen | a | a | a | 0.46 | b | a | b | 0.013*** |
