# Supplementary material for: Mycobiome of Cysts of the Soybean Cyst Nematode Under Long Term Crop Rotation
Source: Front Microbiol. 2018 Mar 16;9:386. doi: 10.3389/fmicb.2018.00386 (PMC5865410; doi:10.3389/fmicb.2018.00386)
Supplement: Table S13 — FUNGuild categories correlated with SCN egg density and EPI. [file Table13.DOCX]

**STable 13.** Guild significantly correlated with SCN egg density and EPI.

| A Guild significantly correlated with SCN egg desntiy | | | | |
| --- | --- | --- | --- | --- |
| Guild | Season | Equation | R square | *P* value |
| Animal Endosymbiont | Spring15 | Y = -0.73 + 0.001X | 0.53 | <0.0001*** |
| Plant Pathogen | Spring15 | Y = 8.67 + -0.001X | 0.27 | 0.006** |
| Animal Endosymbiont | Mid15 | Y = -0.7 + 0.001X | 0.38 | 0.001*** |
| Orchid Mycorrhizal-Plant Pathogen-Wood Saprotroph | Mid15 | Y = -0.03 + 0.00002X | 0.2 | 0.02* |
| Wood Saprotroph | Mid15 | Y = -0.04 + 0.000007X | 0.25 | 0.01* |
| Leaf Saprotroph | Spring16 | Y = -0.03 + 0.00007X | 0.25 | 0.01** |
| B Guild significantly correlated with EPI | | | | |
| Dung Saprotroph-Plant Saprotroph-Soil Saprotroph | Mid16 | Y = -0.06 + 0.12X | 0.45 | 0.02* |
